# International Analgesia, Sedation, and Delirium Practices: a prospective cohort study

**DOI:** 10.1186/s40560-019-0379-z

**Published:** 2019-04-24

**Authors:** Gary D. Owen, Joanna L. Stollings, Shayan Rakhit, Li Wang, Chang Yu, Morgan A. Hosay, James W. Stewart, Fernando Frutos-Vivar, Oscar Peñuelas, Andres Esteban, Antonio R. Anzueto, Konstantinos Raymondos, Fernando Rios, Arnaud W. Thille, Marco González, Bin Du, Salvatore M. Maggiore, Dimitrios Matamis, Fekri Abroug, Pravin Amin, Amine Ali Zeggwagh, Mayur B. Patel

**Affiliations:** 1 Department of Pharmaceutical Services, Vanderbilt University Medical Center, Nashville, TN USA; 2 Critical Illness, Brain Dysfunction, & Survivorship Center, Vanderbilt University Medical Center, Nashville, TN USA; 3 Vanderbilt University School of Medicine, Nashville, TN USA; 4 Departments of Surgery, Neurosurgery, and Hearing & Speech Sciences, Vanderbilt University Medical Center, Nashville, TN USA; 5 Department of Biostatistics, Vanderbilt University Medical Center, Nashville, TN USA; 6 Baylor University, Waco, TX USA; 7 Meharry Medical College, Nashville, TN USA; 8 Hospital Universitario de Getafe, Madrid, Spain; 9 Centro de Investigación Biomédica en red de Enfermedades Respiratorias, Madrid, Spain; 10 Department of Medicine, University of Texas Health, and South Texas Veterans Health Care System, San Antonio, TX USA; 11 Medizinische Hochschule, Hannover, Germany; 12 Hospital Nacional Alejandro Posadas, Buenos Aires, Argentina; 13 University Hospital of Poitiers, Poitiers, France; 14 Clínica Medellín & Universidad Pontificia Bolivariana, Medellín, Colombia; 15 Peking Union Medical College Hospital, Beijing, People’s Republic of China; 16 Università degli Studi G. d’Annunzio Chieti e Pescara, Chieti, Italy; 17 Papageorgiou Hospital, Thessaloniki, Greece; 18 Hospital Fattouma Bourguina, Monastir, Tunisia; 19 Bombay Hospital Institute of Medical Sciences, Mumbai, India; 20 Centre Hospitalier Universitaire Ibn Sina - Mohammed V University, Rabat, Morocco; 21 Center for Health Services Research and Vanderbilt Brain Institute, Vanderbilt University Medical Center, Nashville, TN USA; 22 Surgery Service and Geriatric Research, Education and Clinical Center (GRECC) at the Department of Veterans Affairs Medical Center, Tennessee Valley Healthcare System, Nashville, TN USA

**Keywords:** Delirium, Critical illness, Analgesia, Agitation, Mechanical ventilation

## Abstract

**Background:**

While understanding of critical illness and delirium continue to evolve, the impact on clinical practice is often unknown and delayed. Our purpose was to provide insight into practice changes by characterizing analgesia and sedation usage and occurrence of delirium in different years and international regions.

**Methods:**

We performed a retrospective analysis of two multicenter, international, prospective cohort studies. Mechanically ventilated adults were followed for up to 28 days in 2010 and 2016. Proportion of days utilizing sedation, analgesia, and performance of a spontaneous awakening trial (SAT), and occurrence of delirium were described for each year and region and compared between years.

**Results:**

A total of 14,281 patients from 6 international regions were analyzed. Proportion of days utilizing analgesia and sedation increased from 2010 to 2016 (*p* < 0.001 for each). Benzodiazepine use decreased in every region but remained the most common sedative in Africa, Asia, and Latin America. Performance of SATs increased overall, driven mostly by the US/Canada region (24 to 35% of days with sedation, *p* < 0.001). Any delirium during admission increased from 7 to 8% of patients overall and doubled in the US/Canada region (17 to 36%, *p* < 0.001).

**Conclusions:**

Analgesia and sedation practices varied widely across international regions and significantly changed over time. Opportunities for improvement in care include increasing delirium monitoring, performing SATs, and decreasing use of sedation, particularly benzodiazepines.

**Electronic supplementary material:**

The online version of this article (10.1186/s40560-019-0379-z) contains supplementary material, which is available to authorized users.

## Background

Patient outcomes among critically ill patients are significantly affected by occurrences of pain, agitation, and delirium (PAD). For instance, delirium, a fluctuating disturbance in attention and awareness, has been estimated to occur in up to 80% of mechanically ventilated patients and has been associated with increased cost, length of stay, duration of mechanical ventilation, and mortality, as well as long-term cognitive impairment [1–4]. Given this scope and significance, past and present guidelines have recommended strategies for preventing and treating pain, agitation, and delirium [1, 5]. Guidelines published in 2013 by the Society of Critical Care Medicine include recommendations for adequate analgesia, lighter sedation, performance of spontaneous awakening trials (i.e., daily cessation of sedation and reassessment of sedation needs), and preferential use of non-benzodiazepine sedatives.

Despite evidence supporting guideline recommendations, however, it is unclear how fully and quickly recommendations have been adopted into practice. To date, limited surveys of practitioners and institutions have revealed delayed and incomplete adoption of guidelines along with inflated perceptions of adherence to guidelines and best practices. [6–10].

The objectives of this study were first to characterize trends in pain and sedation strategies over time and across world regions using actual patient care data and second to identify aspects of clinical practice associated with occurrence of delirium in critically ill patients. We hypothesized that practice would vary significantly between year and region and that delirium would be less frequent in 2016 than in 2010.

## Methods

### Study design and population

We performed a retrospective analysis of a multicenter, international, institutional review board (IRB)-approved, prospective cohort study. Data were acquired from the third (March 2010) and fourth (July 2016) International Studies of Mechanical Ventilation (ISMV), which occurred before and after publication of the 2013 PAD guidelines. Conducted every 6 years since 1998, the ISMV primarily investigates the impact of ventilation practices on mortality. Over the course of 1 month, clinical data were collected prospectively on mechanically ventilated patients until discharge, death, or 28 days after admission. The first three ISMV studies have been described previously [11–13], and the fourth ISMV cohort is registered on www.clinicaltrials.gov (NCT02731898).

Included patients in the fourth ISMV cohort must have been admitted to an intensive care unit (ICU) requiring invasive mechanical ventilation (endotracheal tube or tracheostomy) for longer than 12 h or non-invasive mechanical ventilation (bilevel positive airway pressure [BIPAP] or continuous positive airway pressure [CPAP]) for more than 1 h or have been transferred to a participating ICU after already receiving mechanical ventilation. Patients less than 18 years of age and those admitted after elective surgery requiring less than 12 h of invasive mechanical ventilation were excluded.

### Measurements and outcomes

Our outcomes were twofold. First, we analyzed how analgesia and sedation strategies varied across years and regions, including proportion of patient days receiving analgesics and sedatives, choice of sedatives, and performance of spontaneous awakening trials (SATs). Second, we investigated how occurrence of delirium varied by year and region.

Daily use of analgesia and sedation were defined in the ISMV as an infusion utilized for longer than three consecutive hours and are herein described as proportion of total patient days with sedation (referred to as a sedation day) or analgesia. Use of sedative agents are further described as proportion of sedation days receiving each agent. Performance of SATs are described as the proportion of sedation days incorporating a cessation of sedation.

The features of inattentiveness, disorganized thinking, and altered consciousness were employed as general protocol definition for delirium. However, countries participating in this cohort could have used any delirium tool. In addition, level of sedation using Richmond Agitation and Sedation Scale (RASS) was required to be between − 3 and + 4 for a delirium classification (ISMV 4 only; RASS not collected in ISMV 3). Additional collected variables included age, gender, body mass index (BMI), simplified acute physiology score (SAPSII), international region, reason for ventilation, choice of sedative and analgesic, performance of an SAT, use of neuromuscular blockage (NMB), and RASS (ISMV 4 only). Measurements were performed on a daily basis per ISMV study protocol.

### Statistical analysis

Cohorts from 2010 and 2016 were compared overall and for each region. Median and interquartile range (IQR) are presented for continuous variables, while count and proportion (*n*, %) are presented for categorical variables. For clinical practice (e.g., sedation use), differences in proportion of days were calculated and weighted individual proportions were used to calculate standard errors and *p* values to account for varying length of stay and data points for each patient. Pearson’s chi-squared test was used to compare prevalence of delirium. In addition, a multinomial regression model was used to investigate associations of various risk factors with daily development of delirium or coma with normal (i.e., no delirium, no coma) as reference in the 2016 cohort. Model covariates included baseline variables (age, gender, BMI, SAPS II, region), previous day clinical variables (use of propofol, use of benzodiazepines, use of dexmedetomidine, use of analgesia, use of neuromuscular blockers), performance of spontaneous awakening trial, and day of admission.

Statistical analyses were performed using IBM SPSS Statistics Version 24.0 (Armonk, NY: IBM Corp) and statistical software R version 3.3.0 (R Development Core Team Vienna, Austria; https://www.r-project.org/), considering *p* < 0.05 to indicate statistical significance.

## Results

This study evaluated analgesia and sedation practices in 14,281 patients in 6 international regions before and after publication of 2013 PAD guidelines. Demographics were similar between 2010 and 2016 (Table 1) with median ages of 63 and 64 years, respectively, and SAPS II scores of 45 and 44, correlating to an estimated hospital mortality of approximately 35% [14]. Europe was the most represented region with approximately 40% of patients, followed by Latin America (22% in 2010, 26% in 2016), and Asia (17% in 2010, 26% in 2016). The United States (US) and Canada combined region contributed 11% and 5% of patients in 2010 and 2016, respectively. The most common reason for mechanical ventilation was a postoperative status (22% in 2010, 23% in 2016). Sepsis and pneumonia were each the reason for mechanical ventilation in approximately 10% of patients in each year. In addition, there were no differences in length of stay (5 vs. 4 days) or duration of mechanical ventilation (4 days) between groups (2010 vs. 2016).

**Table 1:** Baseline demographics

	2010 *n* = 7323	2016 *n* = 6958
Age	63 (49–74)	64 (50–75)
Male	4571 (62%)	4347 (62%)
BMI	25.5 (22.9–28.9)	25.1 (22.6–28.3)
SAPSII	45 (33–58)	44 (32–58)
Region		
USA/Canada	825 (11.3%)	322 (4.6%)
Europe	2931 (40.0%)	2677 (38.5%)
Africa	133 (1.8%)	169 (2.4%)
Asia	1226 (16.7%)	1816 (26.1%)
Latin America	1576 (21.5%)	1809 (26.0%)
Australia/New Zealand	632 (8.6%)	165 (2.4%)
Reason for ventilation		
COPD exacerbation	336 (4.6%)	323 (4.6%)
Asthma	80 (1.1%)	44 (0.6%)
Other chronic pulmonary disease	87 (1.2%)	116 (1.7%)
Coma^a^		
Metabolic	265 (3.8%)	287 (4.4%)
Overdose/intoxication	211 (3.0%)	197 (3.0%)
Hemorrhagic stroke	470 (6.7%)	434 (6.6%)
Ischemic stroke	214 (3.1%)	199 (3.0%)
Brain trauma	302 (4.3%)	378 (5.7%)
Neuromuscular disease	74 (1.0%)	93 (1.4%)
Acute respiratory failure		
ARDS	256 (3.5%)	284 (4.1%)
Postoperative	1702 (23.2%)	1538 (22.1%)
CHF	399 (5.4%)	346 (5.0%)
Aspiration	188 (2.6%)	167 (2.4%)
Pneumonia	697 (9.5%)	737 (10.6%)
Sepsis	715 (9.8%)	700 (10.6%)
Trauma	347 (4.7%)	296 (4.3%)
Cardiac arrest	473 (6.8%)	443 (6.7%)
Other ARF	400 (5.5%)	261 (3.8%)

Continuous variables reported as median (IQR). Categorical variables reported as *n* (%)

^a^Coma subtype was missing in 341 and 384 patients in 2010 and 2016, respectively

Definitions of abbreviations: *ARDS* acute respiratory distress syndrome, *ARF* acute respiratory failure, *CHF* congestive heart failure, *BMI* body mass index, *SAPS* simplified acute physiology score

The use of analgesia and sedation varied between study years and regions. From 2010 to 2016, the proportion of patient days with opioid infusions increased from 45 to 62% (*p* < 0.001), and the proportion of patient days with sedative infusions (i.e., sedation days) increased from 47 to 58% (*p* < 0.001) (Table 2). Similar increases were seen in all regions.

**Table 2:** Proportion of days utilizing analgesia and sedation

	2010	2016	Difference (95% CI)	*p* value
Analgesia				
Overall	23,854/52803 (45%)	25,017/40612 (62%)	0.164 (0.160 to 0.168)	< 0.001
Europe	11,472/22005 (52%)	10,931/15592 (70%)	0.180 (0.173 to 0.187)	< 0.001
US/Canada	2142/4984 (43%)	1054/1993 (53%)	0.099 (0.083 to 0.115)	< 0.001
Asia	3033/9335 (32%)	4398/10096 (44%)	0.111 (0.103 to 0.119)	< 0.001
Africa	254/869 (29%)	499/912 (55%)	0.255 (0.224 to 0.286)	< 0.001
Australia/New Zealand	1579/3094 (51%)	533/672 (79%)	0.283 (0.259 to 0.307)	< 0.001
Latin America	5374/12516 (43%)	7602/11347 (67%)	0.241 (0.233 to 0.249)	< 0.001
Sedation				
Overall	24,925/52803 (47%)	23,520/40612 (58%)	0.107 (0.102 to 0.112)	< 0.001
Europe	11,773/22005 (54%)	10,468/15592 (67%)	0.136 (0.129 to 0.143)	< 0.001
US/Canada	2439/4984 (49%)	1030/1993 (52%)	0.027 (0.008 to 0.046)	0.003
Asia	3401/9335 (36%)	4507/10096 (45%)	0.082 (0.073 to 0.091)	< 0.001
Africa	281/869 (32%)	500/912 (55%)	0.225 (0.192 to 0.258)	< 0.001
Australia/New Zealand	2016/3094 (65%)	577/672 (86%)	0.207 (0.183 to 0.231)	< 0.001
Latin America	5015/12516 (40%)	6438/11347 (57%)	0.167 (0.158 to 0.176)	< 0.001

The choice of sedative varied between years and regions, as well (Table 3). In 2010, benzodiazepines were the most frequently used sedative in all regions with use being highest in Africa and Latin America (95% of sedation days). From 2010 to 2016, the use of any benzodiazepine decreased from 71 to 55% of sedation days overall (*p* < 0.001). Africa experienced the least change in choice of sedative between 2010 and 2016. Overall, the use of propofol increased from 38 to 41% of sedation days from 2010 to 2016 (*p* < 0.001). By 2016, propofol had become the most frequently used sedative in the US/Canada, Europe, and Australia/New Zealand, while Africa, Latin America, and Asia still utilized benzodiazepines most frequently. In addition, propofol was nearly twice as common in Australia and New Zealand in 2010 compared to other regions. Use of dexmedetomidine increased from 0.8 to 11% overall (*p* < 0.001), with Asia using it most frequently in 2016 (29% of sedation days).

**Table 3:** Proportion of days using sedative agents

Benzodiazepines				
Overall	17,627/24925 (71%)	12,955/23520 (55%)	− 0.156 (− 0.16 to − 0.152)	< 0.001
Europe	7178/11773 (61%)	4939/10468 (47%)	− 0.138 (− 0.145 to − 0.131)	< 0.001
US/Canada	1803/2439 (74%)	372/1030 (36%)	− 0.378 (− 0.397 to − 0.359)	< 0.001
Asia	2725/3401 (80%)	2093/4507 (46%)	− 0.337 (− 0.346 to − 0.328)	< 0.001
Africa	267/281 (95%)	448/500 (90%)	− 0.054 (− 0.069 to − 0.039)	< 0.001
Australia/New Zealand	887/2016 (44%)	122/577 (21%)	− 0.229 (− 0.255 to − 0.203)	< 0.001
Latin America	4767/5015 (95%)	4981/6438 (77%)	− 0.177 (− 0.183 to − 0.171)	< 0.001
Propofol				
Overall	9526/24925 (38%)	9728/23520 (41%)	0.031 (0.027 to 0.035)	< 0.001
Europe	6012/11773 (51%)	6095/10468 (58%)	0.072 (0.064 to 0.08)	< 0.001
US/Canada	829/2439 (34%)	672/1030 (65%)	0.313 (0.294 to 0.332)	< 0.001
Asia	876/3401 (26%)	1380/4507 (31%)	0.049 (0.039 to 0.059)	< 0.001
Africa	15/281 (5%)	34/500 (7%)	0.015 (0.002 to 0.028)	0.023
Australia/New Zealand	1545/2016 (77%)	496/577 (86%)	0.093 (0.069 to 0.117)	< 0.001
Latin America	249/5015 (5%)	1051/6438 (16%)	0.114 (0.108 to 0.12)	< 0.001
Dexmedetomidine				
Overall	196/24925 (0.8%)	2615/23520 (11%)	0.103 (0.101 to 0.105)	< 0.001
Europe	24/11773 (0.2%)	609/10468 (6%)	0.056 (0.053 to 0.059)	< 0.001
US/Canada	5/2439 (0.2%)	84/1030 (8%)	0.08 (0.068 to 0.092)	< 0.001
Asia	14/3401 (0.4%)	1309/4507 (29%)	0.286 (0.28 to 0.292)	< 0.001
Africa	0/281 (0%)	0/500 (0%)	–	–
Australia/New Zealand	32/2016 (1.6%)	59/577 (10%)	0.086 (0.07 to 0.102)	< 0.001
Latin America	121/5015 (2.4%)	554/6438 (9%)	0.062 (0.057 to 0.067)	< 0.001

Proportion of days using each sedative out of the total days when any sedation was received

Between 2010 and 2016, the performance of SATs increased from 20 to 21% of sedation days (*p* < 0.001). The overall increase was driven by the US/Canada which saw performance of SATs increase from 24 to 35% of sedation days (*p* < 0.001). SAT performance increased modestly in Europe from 15 to 18% (*p* < 0.001), stayed relatively flat in Asia, Latin America, and Australia/New Zealand, and decreased in Africa (*p* < 0.001) (Table 4).

**Table 4:** Proportion of days utilizing spontaneous awakening trials

	2010	2016	Difference (95% CI)	*p* value
Overall	4848/24925 (20%)	4963/23520 (21%)	0.017 (0.013 to 0.021)	< 0.001
Europe	1771/11773 (15%)	1892/10468 (18%)	0.03 (0.023 to 0.037)	< 0.001
US/Canada	589/2439 (24%)	356/1030 (35%)	0.104 (0.082 to 0.126)	< 0.001
Asia	1017/3401 (30%)	1361/4507 (30%)	0.003 (− 0.006 to 0.012)	0.521
Africa	92/281 (33%)	77/500 (15%)	− 0.173 (− 0.198 to − 0.148)	< 0.001
Australia/New Zealand	446/2016 (22%)	120/577 (21%)	− 0.013 (− 0.042 to 0.016)	0.360
Latin America	933/5015 (19%)	1157/6438 (18%)	− 0.006 (− 0.015 to 0.003)	0.165

Proportion of days with SAT performed out of the total days when any sedation was received

As shown in Table 5, any occurrence of delirium during admission increased from 7% of patients in 2010 to 9% of patients in 2016 (*p* = 0.007), driven by the US/Canada region, which saw delirium rates double between 2010 and 2016 (17% vs. 36%, *p* < 0.001). Occurrence of delirium also increased in Latin America (5% vs. 10%, *p* < 0.001). There were no significant changes in Europe (6% vs. 6%, *p* = 0.964), Asia (6% vs. 7%, *p* = 0.152), Africa (1% vs. 0%, *p* = 0.440), or Australia/New Zealand (13% vs. 8%, *p* = 0.098).

**Table 5:** Prevalence of delirium during admission

	2010	2016	*p* value
Overall	542/7323 (7%)	600/6958 (9%)	0.007
Europe	182/2931 (6%)	167/2677 (6%)	0.964
US/Canada	136/825 (17%)	115/322 (36%)	< 0.001
Asia	71/1226 (6%)	129/1816 (7%)	0.152
Africa	1/133 (1%)	0/169 (0%)	0.440
Australia/New Zealand	79/632 (13%)	13/165 (8%)	0.098
Latin America	73/1576 (5%)	176/1809 (10%)	< 0.001

Multinomial analysis was consistent with previous literature identifying benzodiazepine use with increased development of delirium and dexmedetomidine use with decreased development of delirium. Full results are shown in the Additional file 1

## Discussion

As hypothesized, the degree of implementation of recommended sedation strategies varied dramatically by region. In accordance with guideline recommendations, use of analgesia increased, performance of SATs increased, and use of benzodiazepines decreased. However, opportunities for improvement include minimizing overall sedation, continuing to decrease benzodiazepine use, and increasing performance of SATs. Contrary to our hypothesis, occurrence of delirium did not change or increased from 2010 to 2016.

Use of non-benzodiazepine sedatives has been associated with decreased ICU length of stay, increased ventilator free days, and decreased incidence of delirium [15–18]. Likewise, performance of daily SATs has been shown to reduce duration of mechanical ventilation and decrease rates of post-traumatic stress disorder after an ICU admission [19, 20]. Bundling such guideline-based practices during ICU admission has recently been shown to decrease mortality [21–23]. Differences in benzodiazepine use between regions may reflect resource constraints, as benzodiazepines cost substantially less than propofol and dexmedetomidine. However, use of non-benzodiazepines and lighter sedation strategies may be cost-effective overall due to improvement in patient outcomes and decreased overall resource utilization [24].

The observed increase in delirium from 2010 to 2016, especially in the US/Canada region may be due to observation bias as awareness and training for identifying delirium increase. The single-digit rates seen in this study conflict with previous estimates of delirium occurring in up to 80% of mechanically ventilated patients [2]. Daily delirium assessment using a validated tool was protocolized, and only a small percentage of data were missing, but it is possible that assessment and documentation of delirium were inconsistent between years, regions, and sites. This highlights the need for education and training for accurate assessment of delirium in clinical practice and studies and to engage in consistent delirium monitoring.

Our study has several strengths. Most notably, this is the first study to our knowledge to assess guideline implementation using actual clinical practice throughout admission. Our findings confirm that implementation of guideline-recommended sedation strategies is incomplete and much lower than previously suggested [6–10]. The large size, international scope, use of daily measures, and broad inclusion criteria all support the strength of the findings. Therefore, we consider the findings to be widely representative and applicable to the care of critically ill adults.

Limitations are largely related to the nature of data collection in the original ISMV studies. Across this unfunded international research collaborative, there was no resource or ability to standardize the execution of the PAD guidelines, despite the multinational, interdisciplinary nature of the original guideline. Our goal was simply to determine how this recommendation translated into actual practice change in the real world. Therefore, not all guideline recommendations were able to be considered using the available data. Likewise, assessments of patient measures were often limited to once daily monitoring, which may not accurately reflect the rapidly changing nature of critical care medicine and delirium. Dosages of medications were not considered, which would have made for a more robust analysis. Finally, quality and accuracy of delirium assessment is unknown and appears lower than expected based on previous studies. However, if delirium rates are underestimated, the findings of our multinomial regression would also likely be underestimated, suggesting that the true association of delirium with previously identified risk factors would be even stronger. Despite these limitations, this study provides considerable insight into trends in sedation practices and implementation of the 2013 PAD guidelines.

These findings highlight how critical care practice has improved and where opportunities still exist. An interprofessional team is critical for addressing all aspects of care and implementing guideline-based care, including minimizing use of sedation and performing daily SATs. The movement of guidelines and evidenced-based practice into routine clinical use requires detailed examination of patient, provider, facility, and policy factors influencing consistent implementation. Despite the strong data supporting SAT and other PAD elements, there are likely weaknesses across knowledge dissemination, resources, leadership, and programmatic quality/process improvement initiatives creating heterogeneity of practices across the world. These unique environment-specific barriers in implementation for PAD guidelines still need to be better defined. More work is needed to address how to implement these best practices. Future directions should include evaluating implementation of other aspects of the guidelines, accounting for cumulative medication doses including intermittent analgesia and sedation use, developing structured team-based approaches, and identifying methods for preventing and treating ICU delirium.

## Conclusions

In these multicenter, international, prospective cohorts of mechanically ventilated adults, we observed substantial differences in sedation strategies between 2010 and 2016, before and after publication of 2013 PAD guidelines. In addition, practices varied widely between regions. In accordance with PAD guideline recommendations, use of benzodiazepines decreased among all regions, though benzodiazepines remained the sedative of choice in Africa, Latin America, and Asia in 2016. Despite increases in the performance of SATs, especially in the US/Canada region, SATs were performed a minority of the time. Occurrence of delirium increased slightly overall possibly due to observation bias and limited delirium monitoring.

## Additional file

Additional file 1: **Table** **1:** Risk factors for daily development of delirium in 2016 cohort. (DOCX 56 kb)

## Abbreviations

BIPAP: Bilevel positive airway pressure
BMI: Body mass index
CPAP: Continuous positive airway pressure
ICU: Intensive care unit
IQR: Interquartile range
IRB: Institutional review board
ISMV: International study of mechanical ventilation
NMB: Neuromuscular blockage
PAD: Pain, agitation, and delirium
RASS: Richmond agitation and sedation scale
SAPSII: Simplified acute physiology score
SAT: Spontaneous awakening trial
US: United States
