# Supplementary material for: International Analgesia, Sedation, and Delirium Practices: a prospective cohort study
Source: J Intensive Care. 2019 Apr 24;7:25. doi: 10.1186/s40560-019-0379-z (PMC6480848; doi:10.1186/s40560-019-0379-z)
Supplement: Supplementary file 1 — Table 1: Risk factors for daily development of delirium in 2016 cohort. (DOCX 56 kb) [file 40560_2019_379_MOESM1_ESM.docx]

# Additional file 1 supplementary appendix

## e-Table 1: Risk Factors for Daily Development of Delirium in 2016 Cohort^a^

| Covariate | Odds Ratio | 95% Confidence Interval |
| --- | --- | --- |
| Age | 0.99 | 0.989-0.994 |
| Female | 1.02 | 0.923-1.121 |
| BMI | 1.00 | 0.995-1.01 |
| SAPS II | 1.02 | 1.022-1.028 |
| Region: Australia/New Zealand | 1.57 | 1.149-2.159 |
| Region: Europe | 1.41 | 1.235-1.599 |
| Region: Latin America | 1.37 | 1.192-1.569 |
| Region: U.S./Canada | 1.31 | 1.027-1.669 |
| Spontaneous Awakening | 0.90 | 0.823-0.982 |
| Propofol | 1.31 | 1.216-1.417 |
| Dexmedetomidine | 0.76 | 0.658-0.882 |
| Benzodiazepines | 1.84 | 1.703-1.983 |
| Opioids | 1.11 | 1.035-1.196 |
| Neuromuscular blocker | 1.79 | 1.596-2.105 |
| Day of admission | 0.95 | 0.937-0.957 |

^a^ Exposure to propofol, dexmedetomidine, benzodiazepines, and opioids was defined as continuous infusions of longer than 3 consecutive hours received the previous day. Africa was excluded since no delirium was reported in 2016.

Definitions of abbreviations: BMI = body mass index , SAPS = simplified acute physiology score

## Study Investigators

Investigators in the Third International Study on Mechanical Ventilation (2010)

*Argentina:*

Coordinators: Fernando Ríos (Hospital Nacional Alejandro Posadas), Damian Violi (Hospital Interzonal General de Agudo Profesor Dr. Luis Guemes, Haedo)

Marisol Rodríguez-Goñi, Roger Lamoglie & Fernando Villarejo (Hospital Nacional Profesor A. Posadas, Buenos Aires); Norberto Tiribelli & Santiago Ilutovich (Sanatorio de la Trinidad, General Mitre); Matías Brizuela & Mariana Monllau (Hospital Tránsito Cáceres de Allende, Córdoba); Fernando Saldarini & Silvina Borello (Hospital General de Agudos donación Francisco Santojanni, Buenos Aires); Alberto Marino & Norberto Tiribelli (Hospital Churruca-Visca, Buenos Aires); Mauricio Vinzio & Karina Bonasegla (Sanatorio de la Trinidad, San Isidro); Julián Hernández & María Belén Yapur (Sanatorio Nuestra Señora del Rosario, Jujuy); María Eugenia González (Hospital Privado de Comunidad, Mar del Plata); Sebastián E. Mare (Sanatorio Dr. Julio Méndez, Buenos Aires); Judith Sagardía & Marco Bezzi (Hospital General de Agudos P. Piñeiro, Buenos Aires); Cecilia Pereyra & Julian Strati (Hospital Interzonal General de Agudo Profesor Dr .Luis Guemes, Haedo); Daniel Vargas & Claudia Diaz (Hospital Pablo Soria, Jujuy); Pablo Gómez & Marcelo Palavecino (Sanatorio Juncal, Temperley); Graciela Elizabeth & Aguilera García, M. Eugenia (Hospital de San Luis); Luis Pablo Cardonnet & Lisandro Betttini (Hospital Provincial del Centenario, Rosario); Hernán Nuñez & Lucas Vallejo (Hospital General de Agudos Juan A. Fernández, Buenos Aires); Fernando Fernández & Jorge Arroyo (Hospital Central, Mendoza); Daniel Duarte & Gerardo Filippa (Hospital Regional Río Grande, Tierra del Fuego); Cayetano Galetti & Hernan Nunia (Sanatorio Allende, Córdoba); Fernando Lambert & Elisa Estenssoro (Hospital Interzonal de Agudos San Martin, La Plata ); Marina Busico & Fernando Villarejo (Clínica Olivos, Vicente López); Javier Horacio Álvarez (Hospital Universitario Austral, Pilar); Alejandro Raimondi & Gustavo Badariotti (Sanatorio Mater Dei, Buenos Aires); Martín Lugaro (Sanatorio Las Lomas, San Isidro); Fernando Lipovestky (Clínica Santa Isabel; Buenos Aires); Alan Javier Zazu & Hugo Capponcelli (Clínica Privada de Especialidades de Villa María); Patricia Vogl & Cristina Orlandi (Hospital Zonal Francisco López Lima, General Roca); Alejandro Gómez & Gustavo Jannello (Sanatorio de los Arcos; Buenos Aires); Alejandro Risso (Sanatorio Otamendi y Miroli, Buenos Aires); Leticia Rapetti & Guillermo Chiappero (Hospital Universitario, Universidad Abierta Interamericana, Buenos Aires); Juan Domingo Fernández (Hospital Regional de Comodoro Rivadavia, Chubut); Rodrigo E. Gómez-Paz (Hospital Español, Buenos Aires); Marcos Juan Zec & Pascual Valdez (Hospital General de Agudos Dalmacio Vélez Sársfield, Buenos Aires); Jorgelina Guyon, Ariel Chena (Hospital Lagomaggiore, Mendoza); Sergio Lasdica (Hospital Municipal Coronel Suárez, Buenos Aires); Martin Deheza, Schimdt Alejandra (Hospital General de Agudos Bernardino Rivadavia, Buenos Aires); Francisco Criado (Hospital Naval Puerto Belgrano, Bahía Blanca); Norma Beatriz Márquez (Policlínico Atlántico del Sur, Ríos Gallegos); Pablo Desmery & José Luis Scapellato (Sanatorio Anchorena, Buenos Aires); Gonzalo Javier Ríos & Cristian Casabella (Clínica Bazterrica, Buenos Aires)

*Australia*:

Coordinators: Jasmin Board & Andrew Davies (Alfred Hospital, Melbourne)

Andrew Bersten, Elisha Matheson & Amy Waters (Flinders Medical Center, Adelaide); John Santamaria & Jennifer Holmes (St Vincent's Hospital, Melbourne); Cartan Costello, Manoj K Saxena & John Myburgh (St George Hospital, Sydney); Ellen Kinkel & Forbes McGain (The Western Hospital, Melbourne); Claire Cattigan & Allison Bone (Barwon Health, Geelong Hospital, Geelong); Ian Seppelt, Leonie Weisbrodt & Cheryl Cuzner (Nepean Hospital, Sydney); Christopher MacIsaac, Deborah Barge & Tania Caf (Royal Melbourne Hospital, Melbourne); Cameron Knott & Graeme Duke (The Northern Hospital, Melbourne); Imogen Mitchell, Helen Rodgers, Rachel Whyte & Elisha Fulton (Canberra Hospital, Canberra); Jasmin Board, Andrew Davies & Alistair Nichol (Alfred Hospital, Melbourne); Hergen Buscher, Priya Nair & Claire Reynolds (St Vincent's Hospital, Sydney); Simon JG Hockley, Ian Moore & Katherine Davidson (Calvary Wakefield Hospital, Adelaide), David Milliss, Raju Pusapati & Helen Wong (Concord Hospital, Sydney); Jason Fletcher & Julie Smith (Bendigo Hospital, Bendigo); Paul Goldrick, Dianne Stephens & Jane Thomas (Royal Darwin Hospital, Darwin); Anders Aneman, Sutrisno Gunawan & Tom Cowlam (Liverpool Hospital, Liverpool); George Lukas & Rick McAllister (Royal Hobart Hospital, Hobart); Minka Springham, Joanne Sutton & Jeff Presneill (Mater Health Services, Brisbane); Tony Sutherland & Dianne Hill (Ballarat Health Services, Ballarat); Howard Connor, Jenny Dennett & Tim Coles (Central Gippsland Hospital, Sale).

*Bolivia:*

Coordinator: Freddy Sandy (Hospital Obrero No.1, La Paz)

Sando Chavarria & Marcelo Choque (Hospital Obrero No.1, La Paz), Ronald Pairumani & Juan Guerra (Instituto Gastroenterológico, La Paz)

*Brazil*:

Coordinator: Marco Antônio Soares Reis (Hospital Universitário São José, Belo Horizonte)

José Carlos Versiani (Hospital Madre Teresa, Belo Horizonte); Eduardo Fonseca Sad (Hospital Luxemburgo , Belo Horizonte); Maria Aparecida Braga (Hospital Dia e Maternidade Unimed-BH; Belo Horizonte); Dinalva Aparecida Gomes (Hospital Vera Cruz, Belo Horizonte); Fernando Antônio Botoni (Hospital de Pronto Socorro Risoleta Tolentino Neves, Belo Horizonte); Maurício Meireles Góes (Hospital da Baleia, Belo Horizonte); Frederico Costa Val Barros (Hospital da Polícia Militar, Belo Horizonte); Rogério de Castro Pereira (Hospital Felício Rocho, Belo Horizonte); Hugo Urbano (Hospital Vila da Serra, Belo Horizonte); Valéria de Carvalho Magela (Hospital Santa Rita, Contagem); Aline Camile Yehia (Hospital Júlia Kubitschek, Belo Horizonte); Bruno Bonaccorsi Fernandino (Hospital São Francisco-Setimig, Belo Horizonte); Marco Antônio Ribeiro Leão (Hospital São João de Deus, Divinópolis); Rovílson Lara (Hospital Arnaldo Gavazza Filho, Ponte Nova); Rovílson Lara (Hospital São João Batista, Viçosa); Rubens Altair Amaral de Pádua (Hospital Vaz Monteiro, Lavras); Janine Dias Alves (Santa Casa de Misericórdia de Ouro Preto, Ouro Preto); Aloísio Marques do Nascimento (Hospital Nossa Senhora das Graças, Sete Lagoas); Bruno do Valle Pinheiro (Hospital Universitário da Universidade Federal de Juiz de Fora, Juiz de Fora); Carlos Alberto Studart Gomes (Hospital de Messejana, Fortaleza); Marcelo Alcântara Holanda (Hospital Universitário Walter Cantídio, Fortaleza); Frederico Rodrigues Anselmo (Hospital Nossa Senhora Aparecida, Belo Horizonte).

*Canada*:

Coordinator: Niall Ferguson (Mount Sinai Hospital & University Health Network, Toronto)

Neill Adhikari, Damon Scales, Robert Fowler, Cheromi Sittambalam, Mehar-Un-Nisa Raja & Nicole Marinoff (Sunnybrook Health Science Centre, Toronto); Lauralyn McIntryre, Shawna Reddie, Laura Jones, & Irene Watpool (Ottawa Hospital, Ottawa); Jeffrey Singh & Madison Dennis (Toronto Western Hospital, Toronto); Andrea Matte, Marc Lipkus & Ryan Albert (Toronto Hospital Western Division, Toronto); Andrew Steel & Emily Stern (Toronto General Hospital, Toronto); Michael Miletin, Antonio Raso, & Robyn Klages (William Osler Health Science Centre, Brampton); Jan Friedrich, Orla Smith & Laura Wilson (St Michael’s Hospital, Toronto); Deborah Cook & Mark Bailey (St Joseph’s Hospital, Hamilton); Sangeeta Mehta, Stephen Lapinsky, Hannah Mathers, Cheryl Ethier, Stephanie Lubchansky & Samer Haj-Bakri (Mount Sinai Hospital, Toronto); Dietrich Henzler, & Lisa Julien (Queen Elizabeth II Health Sciences Centre, Halifax).

*Chile*:

Coordinator: Luis Soto-Román (Instituto Nacional del Tórax, Santiago)

Juan Carlos Maurelia (Hospital de Copiapo); César Antonio Maquilon (Instituto Nacional del Tórax, Santiago); Luis Soto-Germani (Hospital de Coquimbo).

*China*:

Coordinator: Bin Du (Peking Union Medical College Hospital, Beijing)

Yan Kang & Bo Wang (West China Hospital, Chengdu); Fachun Zhou & Fang Xu (Chongqing Medical University 1st Hospital, Chongqing); Haibo Qiu & Yi Yang (Southeast University Zhongda Hospital; Nanjing); Qingyuan Zhan & Bing Sun (Beijing Chaoyang Hospital, Beijing); Zhenjie Hu & Bin Yu (Hebei Medical University 4th Hospital, Shijiazhuang); Xi Zhu & Yu Bai  (Peking University 3rd Hospital; Beijing); Gang Li & Yi Li (Sino-Japanese Friendship Hospital, Beijing); Geng Zhang & Jianbiao Meng (Zhejiang Tongde Hospital; Hangzhou); Xiaobo Huang & Hong Pu (Sichuan Provincial Hospital; Chengdu); Bin Du & Daxing Yu (Peking Union Medical College Hospital, Beijing); Chuanyun  Qian & Wei Zhang (Kunming Medical College 1st Hospital, Kunming); Yongjie Yin & Debiao Song (Jilin University 2nd Hospital, Changchun); Yunxuan Yue & Zhengxuan Lv (Kunming City 3rd People's Hospital, Kunming); Chengmin Yu & Qunmei Yao (Yunnan Chuxiong People's Hospital; Chuxiong); Xue Wang (Xi'an Jiaotong University 1st Hospital; Xi’an); Yuan Xu & Wei He (Beijing Tongren Hospital, Beijing); Mian Chen & Zhihua Hu (Hainan Medical College Hospital; Haikou); Dongpo Jiang & Jian Huang (Daping Hospital, Chongqing); Wei Yu (Yantai Yuhuangding Hospital; Yantai); Juanxian Gu  (Zhejiang Haining People's Hospital, Naining); Yangong Chao (Beijing Huaxin Hospital, Beijing); Zhixiang Li (Fengrun District People's Hospital; Tangshan); Zhicheng Zhang (PLA Navy General Hospital, Beijing); Wanxia Li (Nanchang University 2nd Hospital, Nanchang); Zhenyang He (Hainan Provincial People's Hospital, Haikou); Jianguo Li & Chang Liu (Wuhan University Zhongnan Hospital, Wuhan); Tiehe Qin & Shouhong Wang (Guangdong General Hospital, Guangzhou); Feng Li (Nantong 1st People's Hospital, Nantong); Jun Jin & Jianhong Fu (Suzhou University 1st Hospital, Suzhou); Hongyang Xu  (Wuxi Municipal People's Hospital, Wuxi); Hongyuan Lin & Jianying Guo (PLA 304 Hospital, Beijing); Yalin Liu & Jinghua Wang (Beijing Hospital, Beijing); Maoqin Li & Jiaqiong Li (Xuzhou Central Hospital, Xuzhou); Lei Chen (Sun Yet-Sen University 6th Hospital, Guangzhou); Qing Song & Liang Pan (PLA General Hospital, Beijing); Xianyao Wan & Jiuzhi Zhang (Dalian Medical University 1st Hospital, Dalian); Weihai Yao & Yuhong Guo (Beijing TCM Hospital, Beijing); Pang Wing Yan ( Prince Margaret Hospital, Hong Kong); Kelly Choy (Queen Elizabeth Hospital, Hong Kong); Kwan Ming Chit (Pamela Youde Nethersole Eastern Hospital, Hong Kong); Patricia Leung (Prince Of Wales Hospital, Hong Kong); Chau Chin Man (North District Hospital, Hong Kong)

*Colombia*:

Coordinator: Marco González (Clínica Medellín & Universidad Pontificia Bolivariana, Medellín)

Ricardo Buitrago (Clínica Shaio, Bogotá); Marcela Granados (Clínica Fundación Valle Lili, Cali); Guillermo Ortiz (Hospital Santa Clara, Bogotá); Cesar Enciso (Grupo Cimca Hospital San José, Bogotá); Mario Gómez  (Grupo Cimca Hospital San José & Clínica Fundadores, Bogotá); Bladimir Alejandro Gil (Clínica Las Américas, Medellín); Juan Pablo Sedano, Luis Fernando Castro Castro (Centro Medico Imbanaco, Cali); Carlos Alberto Acosta (Hospital Federico Lleras Acosta, Ibague); Marco Gonzalez A.(Hospital San Rafael, Itagui); Francisco Molina (Clínica Universitaria Bolivariana, Medellín); Camilo Pizarro (Fundación cardiovascular colombiana, Bucaramanga); Mario Villabon (Grupo Cimca Hospital de Suba, Bogota); Carmelo Dueñas (Nuevo Hospital Bocagrande, Cartagena de Indias); Carlos Andrés Díaz (Hospital General de Medellín); Nelson Fonseca (Corbic, Medellín); Rubén Camargo (Clínica General del Norte, Barranquilla), Juan David Uribe (Clínica Cardiovascular, Medellín)

*Denmark*:

Coordinator: Hans-Henrik Bülow (Holbak)

Simona Beniczky & Jens Brushoj (Naestved); Mikkel Præst; (Nykobing Falster ); Birgitte Viebaek & Sine Wichman; (Roskilde); Anette Mortensen (Holbak); Susanne Andi Iversen (Slagelse); Bo Broberg (Koge)

*Dominican Republic*:

Edgard Luna (Hospital Universitario José María Cabral y Báez, Santiago)

*Ecuador*:

Coordinator: Manuel Jibaja (Hospital Militar de Quito)

Leonardo Pazmiño, Katty Trelles & Fabricio Picoita (Hospital Eugenio Espejo, Quito); Gustavo Paredes & Vanesa Ramírez (Hospital Enrique Garcés, Quito); Guillermo Falconí, Cristian Cevallos & Boris Villamagua; (Hospital Carlos Andrade Marín, Quito); Marco Escobar & Freddy Sánchez (Hospital de la Policía, Quito); Miguel Llano & Miguel Lazcano (Hospital General de las Fuerzas Armadas, Quito); Ramiro Puetate & José Miguel Guerrero (Hospital Pablo Arturo Suárez, Quito); Mijail Játiva & Myriam Montalvo (Hospital de los Valles, Quito); Franklin Villegas (Hospital Metropolitano, Quito); Luis González Zambrano, Ronnie Mantilla, Gina Quinde, Andrea Gimenez & Luis Gonzalez Mosquera (Hospital Luis Vernaza, Guayaquil); Henry Caballero & María Fernanda García (Hospital de SOLCA, Quito); Marcelo Ochoa, Soraya Puertas & Jackeline Coello (Hospital José Carrasco Arteaga, Cuenca); Mario Acosta (Hospital San Vicente de Paul, Ibarra).

*Egypt*:

Medhat Soliman (Cairo University Hospitals, Cairo)

*France*:

Coordinator: Arnaud W. Thille (CHU Henri Mondor, Créteil)

Achille Kouatchet & Alain Mercat (CHU d’Angers); Laurent Brochard (CHU Henri Mondor, Créteil); François Collet (Centre Hospitalier De Saint-Malo); Guillaume Marcotte (Hôpital Édouard Herriot, Lyon); Pascal Beuret (Centre Hospitalier De Roanne); Jean-Christophe M. Richard, Gaëtan Bedunaeu, Pierre-Gildas Guitard & Fabien Soulis (CHU Charles Nicolle, Rouen); Frédéric Bellec (Centre Hospitalier De Montauban); Philippe Berger (Centre Hospitalier de Châlons en Champagne); Dorothée Carpentier, Benoit Veber (CHU Charles Nicolle, Rouen); Salem Ould Zein, Géraldine Dessertaine (CHU De Grenoble); C. Canevet (Hôpital d’Armentières); Fabien Grelon (Centre Hospitalier Du Mans).

*Germany*:

Coordinator: Konstantinos Raymondos (Medizinische Hochschule Hannover)

Rolf Dembinski & Rolf Rossaint (Universitaetsklinikum Aachen); Steffen Weber-Carstens (Charité Universitaetsklinikum, Berlin); Christian Putensen (Universitaetsklinikum Bonn); Maximillian Ragaller (Universitaetsklinikum Carl Gustav Carus, Dresden); Michael Quintel (Universitaetsklinikum der Georg-August-Universitaet, Goettingen); Winfried Schubert (Carl-Thiem-Klinikum Cottbus gGmbH); Thomas Bein & Heinrich Paulus (Klinikum der Universitaet Regensburg); Walter Brandt (Universitaetsklinikum, Magdeburg); Lutz Pfeiffer & Silke Frenzel (Hufeland Klinikum GmbH, Mühlhausen); Thoralf Kerner & P. Kruska (Allgemeines Krankenhaus Harburg, Hamburg); Leila Eckholt & Joachim Hartung (Vivantes Krankenhaus am Urban, Berlin-Kreuzberg); Harald Fritz & Monika Holler (Staedtisches Krankenhaus Martha-Maria Halle-Doelau gGmbH); Johannes Busch & Andreas Viehöfer (Evangelisches Waldkrankenhaus Bad Godesberg gGmbH, Bonn); Jens Buettner (Evangelisches Krankenhaus Elisabethenstift gGmbH, Darmstadt); Jörn Schlechtweg & Achim Lunkeit & Roland Schneider (Klinikum Bad Salzungen GmbH); Maria Wussow & Nils Marquardt & Christian Frenkel (Staedtisches Klinikum Lueneburg); Falk Hildebrandt (Dietrich-Bonhoeffer Klinikum Neubrandenburg); Tumbass Volker & Thomas Lipp (Ermstalklinik Bad Urach); Cezar Mihailescu & Thomas Moellhoff (Katholische Stiftung Marienhospital Aachen); Thomas Steinke (Universitaetsklinikum der Martin-Luther-Universitaet Halle-Wittenberg); Oliver Franke & Marcus Ruecker (Lungenklinik Heckeshorn, Berlin); Markus Schappacher & Steffen Appel (Ermstalklinik Staedtisches Krankenhaus Sindelfingen); Heinz Kerger (Evangelisches Diakoniekrankenhaus, Freiburg); Andreas Schwartz (Bundeswehrkrankenhaus Ulm); Jan Dittmann & Jörg Haberkorn (Georgius-Agricola-Klinikum Zeitz); Wolfgang Baier (St. Nikolaus-Stiftshospital GmbH, Andernach); Walter Seyde (Staedtisches Klinikum Wolfenbuettel)

*Greece*:

Coordinator: Dimitros Matamis (Papageorgiou Hospital, Thessaloniki)

Eleni Antoniadou (Gennimata Hospital, Thessaloniki); Pertsas Evangelos (Agios Pavlos General Hospital, Thessaloniki); Maria Giannakou (Ahepa Hospital, Thessaloniki).

*Hungary*:

Zoltan Szentkereszty (Kenezy Hospital, Debrecen); Zsolt Molnar (University of Szeged)

*India*:

Coordinator: Pravin Amin (Bombay Hospital Institute of Medical Sciences, Mumbai)

Farhad N. Kapadia (Hinduja Hospital, Mumbai); Nagarajan Ramakrishnan (Apollo Hospitals, Chennai); Deepak Govil (Artemis Health Institute, Gurgaon Haryana);Anitha Shenoy & Umesh G (Kasturba Medical College, Manipal); Samir Sahu, (Kalinga Hospital, Odisha); Sheila Nainan Myatra (Tata Memorial Hospital, Mumbai); Subhash Kumar Todi (AMRI Hospitals Kolkata , West Bengal); Sanjay Dhanuka (CHL Apollo Hospital, Indore); Mayur Patel (Saifee Hospital, Mumbai); P Samaddar( Tata Main Hospital, Jamshedpur); Dhruva Chaudhry (PGIMS, Rohtak); Vivek Joshi & Srinivas Samavedam (CARE Hospitals, Surat), Ankur Devendra Bhavsar (Spandan Multispeciality Hospital, Vadodar); Prachee Sathe (Ruby Hall Clinic, Pune); Sujoy Mukherjee (Calcutta medical research institute (CMRI), Kolkat)

*Italy*:

Coordinator: Salvatore Maurizio Maggiore (Policlinico "Agostino Gemelli", Università Cattolica Del Sacro Cuore, Roma)

Francesco Idone & Federica Antonicelli (Policlinico "Agostino Gemelli", Università Cattolica Del Sacro Cuore, Roma); Paolo Navalesi, Rosanna Vaschetto & Arianna Boggero (Ospedale Maggiore Della Carità, Università Del Piemonte Orientale "Amedeo Avogadro", Novara); Rosalba Tufano, Michele Iannuzzi & Edoardo De Robertis (Ospedale Policlinico "Federico II", Università Di Napoli, Napoli); Romano Tetamo & Andrea Neville Cracchiolo (Ospedale "Arnas Civico, Di Cristina, Benfratelli", Palermo), Antonio Braschi, Francesco Mojoli & Ilaria Curro' (ICU 1, Fondazione IRCCS Policlinico “S. Matteo”, Università Di Pavia, Pavia); Mirko Belliato, Chiara Verga & Marta Ferrari (ICU 2, Fondazione IRCCS Policlinico “S. Matteo”, Università Di Pavia, Pavia); Erika Mannelli, Valerio Mangani & Giorgio Tulli (Ospedale "San Giovanni Di Dio", Firenze); Francesca Frigieri & Armando Pedulla' (Ospedale "Santa Maria Annunziata", Firenze); Monica Rocco, Giorgia Citterio & A. Di Russo (Policlinico "Umberto I", Università La Sapienza, Roma); Gaetano Perchiazzi & Loredana Pitagora (Ospedale Policlinico, Università Di Bari, Bari); Antonio Pesenti & Michela Bombino (Ospedale "San Gerardo", Università Di Milano Bicocca, Monza); Davide Chiumello, Federica Tallarini & Serena Azzari (Fondazione IRCCS “Cà Grande” Ospedale Maggiore Policlinico, Università Di Milano, Milano); Antonina Pigna, Ivano Aprile & Marco Adversi (Policlinico Universitario "S. Orsola-Malpighi", Bologna); Antonio Corcione, Marianna Esposito & Annunziata Mattei (Ospedale "V. Monaldi", Napoli); Vito Marco Ranieri, Rosario Urbino & Ilaria Maria Mastromauro (Ospedale "San Giovanni Battista - Molinette", Università Di Torino, Torino); Antonino Giarratano, Maurizio Raineri Santi & Ambrogio Sansone (Policlinico "P. Giaccone", Università Di Palermo, Palermo)

*Japan*:

Toru Katani (Tokyo Women's Medical University)

*Korea*:

Coordinator: Younsuck Koh (Asan Medical Center, University of Ulsan, Seoul)

Moo Suk Park (Hospital Severance, Yonsei University Health System, Seoul); Je Hyeong Kim (Hospital Korea University of Ansan); Kyung Chan Kim (Hospital Catholic University of Daegu); Hye Sook Choi (Hospital Dongguk University of Gyeongju); Yun Seong Kim (Hospital Pusan National University of Yangsan); Jin Hwa Lee (Hospital Ewha Womans University Mokdong, Seoul); Myung-Goo Lee (Hospital Chuncheon Sacred Heart Hospital, Hallym University Medical Center, Chuncheon); Won-Yeon Lee (Hospital Yonsei University Wonju Christian, Wonju); Jin Young An (Hospital Chungbuk National University, Chenogiu); Gee Young Suh (Samsung Medical Center, Sungkyunkwan University, Seoul); Ki-Suck Jung (Hallym University Medical Center, Anyang)

*Mexico*:

Coordinator: Asisclo J Villagómez Ortiz (Hospital Regional 1° de Octubre, ISSSTE, México DF)

César Cruz Lozano (Hospital Regional de Pemex, Ciudad Madero); Zalatiel Maycotte Luna (Hospital Ángeles de las Lomas, México DF); José Francisco López Baca (Hospital Regional de Zona No.1 del IMSS, México DF); José Elizalde (Instituto Nacional De Ciencias Médicas y Nutrición Salvador Zubirán, México DF); Guillermo Cueto Robledo (Hospital General de México, México DF); Mario Alonso Treviño Salinas (Hospital Universitario de Nuevo León 'Dr. Eleuterio González', Nuevo León); Ricardo Martinez Zubieta (Hospital Español de México, Miguel Hidalgo); Claudia Olvera-Guzman & Marco Montes De Oca (Centro Médico ABC , México DF), Silvio A. Ñamendys-Silva (Instituto Nacional de Cancerología, México DF); José Salvador Martínez Cano (Centenario Hospital Miguel Hidalgo , Aguascalientes); Jose Angel Baltazar Torres (Umae Hospital De Especialidades Dr. Antonio Fraga Mouret , México DF); Gustavo Morales Muñoz (Hospital Regional de Alta Especialidad de la Mujer, Villahermosa); Antonio Villa Delgado (Hospital Mérida Yucatán; Mérida); Javier Ladape Martinez (Hospital Juárez de México, Mexico DF).

*Morocco*:

Coordinator: Amine Ali Zeggwagh (Hôpital Ibn Sina, Rabat)

Tarek Dendane (Hôpital Ibn Sina, Rabat); Abderrahim Azzouzi (Hôpital Ibn Sina, Rabat); Ahmed Sbihi (Hôpital Ibn Sina, Rabat); Wajdi Maazouzi & Mourad Amor (Hôpital des Specialites, Rabat); Charki Haimeur (Hôpital Militaire D’Instruction Mohamed V, Rabat).

*Netherlands*:

Coordinator: Michael A. Kuiper (Medical Center Leeuwarden (MCL), Leeuwarden)

Matty Koopmans (Medical Center Leeuwarden (MCL), Leeuwarden); Uli Strauch, Dennis Bergmans & Serge Heines (Universitair Medisch Centrum Maastricht, Maastricht); Sylvia den Boer (Spaarneziekenhuis, Hoofddorp); Bas M. Kors (Kennemer Gasthuis, Haarlem); Peter van der Voort (Onze Lieve Vrouwe Gasthuis (OLVG), Amsterdam); Paul J. Dennesen (Medisch Centrum Haaglanden, Den Haag); Bert Beishuizen, Ingrid van den Hul, Erna Alberts, Harry PPM Gelissen & Eduard Bootsma (Vrije Universiteit Medisch Centrum (VUMC), Amsterdam); Auke Reidinga (Tjongerschans Ziekenhuis, Heerenveen).

*New Zealand*:

Coordinator: Jasmin Board & Andrew Davies (Alfred Hospital, Melbourne)

Kim Heus, Diane Mackle & Paul Young (Wellington Hospital, Wellington); Rachael Parke, Eileen Gilder & Jodi Brown (CVICU Auckland City Hospital, Auckland); Lynette Newby & Catherine Simmonds (DCCM Auckland City Hospital, Auckland); Jan Mehrtens & Seton Henderson (Christchurch Hospital, Christchurch); Tony Williams, Judi Tai & Chantal Hogan (Middlemore, Auckland); Mary La Pine, John Durning & Sheree Gare (Waikato, Hamilton); Troy Browne, Shirley Nelson & Jennifer Goodson (Tauranga Hospital, Tauranga).

*Panama*:

Julio Osorio (Hospital Rafael Hernández, Chiriquí)

*Peru*:

Coordinator: Chabu Coronado

Rollin Roldán Mori (Hospital Edgardo Rebagliatti Martins, Lima); Rosa Luz López Martínez (Hospital Guillermo Almenara Irigoyen, Lima)

*Poland*:

Adam Mikstacki & Barbara Tamowicz (Karol Marcinkowski University Of Medical Sciences, Poznan).

*Portugal*:

Coordinator:  Rui Moreno (Hospital de Santo Antonio dos Capuchos, Centro Hospitalar de Lisboa Central, E.P.E., Lisboa)

Eduardo Almeida (Hospital Garcia de Orta, Almada); Joana Silvestre (Centro Hospitalar de Lisboa Central); Heloisa Castro, Irene Aragão & Susana Alves Ferreira (Centro Hospitalar Do Porto - Hospital Geral De Santo António); Nelson Barros (Centro Hospitalar Trás-Os-Montes E Alto Douro, Vila-Real); Filomena Faria (Ipo); Carlos André Correia Casado (Hospital Da Luz); Fausto Fialho Moura (Hospital de Cascais); Paulo Marcal (Hospital De São Sebastião); Ricardo Matos (Hospital de Santo Antonio dos Capuchos, Centro Hospitalar de Lisboa Central, E.P.E., Lisboa); António Alvarez (Centro Hospitalar de Lisboa Norte)

*Russian Federation*:

Coordinator: Edward Nicolayenko (Hospital No.1, Moscow)

Mikhail Kirov (Hospital No.1, Arkhangelsk); Andrey Yaroshetskiy (Hospital No.7, Moscow); Andrei Piontek (Hospital No.14, Ekaterinburg); Valery Subbotin (National Institute Of Surgery Named After A.V. Vishnevskij, Moscow)

*Saudi Arabia*:

Yaseen Arabi, Olivia Dulfo, Chafrina Marie Olay, Edgardo E. Tabhan (King Saud Bin Abdulaziz University for Health Sciences, Riyadh)

*Spain*:

Coordinator: Nicolas Nin (Hospital Universitario de Getafe, Madrid)

Alfonso Muñoz & César Aragón (Hospital Carlos Haya, Málaga); Ana Villagrá (Corporación Sanitaria y Universitaria Parc Taulí, Sabadell); Ainhoa Rosselló, & Joan Maria Raurich (Hospital Universitario Son Espasses, Mallorca); María Garitacelaya, Miguel Ángel González-Gallego & Francisco Ortuño (Hospital Clínico Universitario San Carlos, Madrid); Miguel Fernández-Vivas (Hospital Virgen de la Arrixaca, Murcia); David Freire (Hospital Juan Canalejo, A Coruña); Francisco Guerrero & Francisco Manzano (Hospital Virgen de las Nieves, Granada); Juan Carlos Sotillo (Hospital Universitario Gregorio Marañón, Madrid); Alejandra Bustos (Hospital de Torrevieja); Alfredo Padrón, Pedro Rosas, Rafael Morales & Liliana Caipe (Hospital Doctor Negrín, Las Palmas de Gran Canaria); Maurizio Bottiroli (Hospital de la Santa Creu i Sant Pau, Barcelona); José María Nicolás (Hospital Clinic-IDIBAPS, Barcelona); Marta Ugalde (Hospital de Cruces, Barakaldo); Javier Ruiz (Hospital Sagrado Corazón, Barcelona); Lucia Capilla (Hospital Morales Meseguer, Murcia); Guillermo Muñiz (Hospital Central de Asturias, Oviedo); Jesús Sánchez-Ruiz (Hospital General de Jerez de la Frontera); Javier Cebrián, Begoña Balerdi, Elena Parreño & Alvaro van Bommel (Hospital Universitario La Fe, Valencia); César Pérez-Calvo (Fundación Jiménez Díaz, Madrid); Irene Dot (Hospital del Mar, Barcelona); Javier Blanco (Complejo Hospitalario de Ciudad Real); Raquel Manzanedo & José J. Blanco (Hospital Insular de Gran Canaria); Daniel Fontaneda, Raúl González & Javier Díaz Domínguez  (Complejo Hospitalario de León); Alfonso Moreno (Hospital San Pedro. Logroño); Antonio Reyes & Ian Carrasco (Hospital de la Princesa, Madrid); Itziar Mintegui, Rosa Sebastián & Javier García-Alonso, (Complejo Hospitalario de Donostia); Carolina Lorencio & Josep Maria Sirvent ( Hospital Universitario Dr.Josep Trueta, Girona); Patricia Jimeno (Hospital General de Segovia); Miguel León (Hospital Arnau de Vilanova, Lleida); Pedro Galdos (Hospital Universitario Puerta de Hierro, Majadahonda); Nuria Alonso (Hospital Universitario Nuestra Santa María del Rosell, Cartagena); Julia López-Díaz (Hospital Universitario La Paz, Madrid); María Victoria de la Torre, Jorge Vidal Hernández & Nicolás Zamboschi (Hospital Universitario Virgen de la Victoria, Málaga); Francisco Lucena (Hospital Universitario de Valme, Sevilla); Gemma Rialp (Hospital Son Llatzer, Mallorca); Raquel Montoiro (Hospital Clínico Lozano Blesa, Zaragoza); Victoria Goñi, María Ángeles Pena & Antonio Maestre (Hospital Virgen del Rocío, Sevilla); Marc Fabra, Jacinto Baena & Eva Benveniste (Hospital Germans Trias I Pujol, Badalona); Susana Temprano (Hospital 12 de Octubre, Madrid); Jesús Sánchez (Hospital de Rio Hortega, Valladolid); Carmen Campos (Hospital Universitario Dr.Peset, Valencia); Sara Cabañes (Hospital Santiago Apóstol, Vitoria); María Elena Pérez-Losada & José Claudio Leo (Complejo Hospitalario de Salamanca); Enrique Piacentini (Hospital Mutua de Terrassa); María del Carmen de la Torre (Hospital de Mataró); Laura Álvarez-Montero, Fernando Sánchez (Hospital Xeral Calde, Lugo); Antonio Viñuales (Hospital Lluis Alcanyis, Xàtiva); Bernabé Álvarez (Hospital General de Alicante); Javier Castañeda (Hospital Clínico de Valladolid); Ángela Alonso (Hospital de Fuenlabrada); María Isabel Ruiz (Complejo Hospitalario de Jaén); Pedro Jesús Domínguez (Hospital Juan Ramón Jiménez, Huelva); Marcos Delgado (Complejo Hospitalari de Manresa); Eugenio Palazón (Hospital Universitario Reina Sofía, Murcia); Antonio García-Jiménez (Hospital Arquitecto Marcide, Ferrol); Rosa Álvaro (Hospital de La Plana, Castellón); Clara Laplaza, Eva Regidor & Enrique Maraví (Complejo Hospitalario de Navarra, Pamplona); José María Quiroga (Hospital de Cabueñes, Gijón); Amalia Martínez de la Gandara (Hospital Infanta Leonor, Madrid); Cecilia Carbayo (Hospital Torrecárdenas, Almería); María Luisa Navarrete (Hospital San Juan, Alicante); Manuel Valledor & Raquel Yano (Hospital San Agustín, Avilés); José María Gutiérrez (Hospital General de Albacete); Javier González-Robledo (Hospital Clínico de Salamanca); Amparo Ferrandiz, Alberto Belenguer & Lidón Mateu (Hospital General de Castellón); Laura Sayagues (Complejo Hospitalario de Santiago de Compostela); María José Tolón (Hospital Royo Vilanova, Zaragoza); Nieves Franco (Hospital de Móstoles); Elena Gallego (Hospital San Pedro de Alcántara, Cáceres); Félix Lacoma (Hospital Quirón, Madrid); Patricia Albert (Hospital del Sureste, Arganda); Vicente Arraez (Hospital Universitario General, Elche); Mar Gobernado (Hospital General de Soria); Susana Moradillo (Hospital Río Carrión, Palencia); Carolina Gímenez-Esparza (Hospital de la Vega Baja, Orihuela); Teresa Sánchez de Dios (Complejo Hospitalario Montecelo, Pontevedra); Carlos Marian Crespo (Hospital General de Guadalajara); Cecilia Hermosa, Federico Gordo (Hospital del Henares, Coslada); Genis Carrasco (Hospital SCIAS, Barcelona); María Ángeles Alonso (Trauma ICU, Hospital 12 de Octubre, Madrid); Alejandro Algora (Fundación Hospital Universitario de Alcorcón, Madrid); Raúl de Pablo (Hospital Príncipe de Asturias, Alcalá de Henares); Sofía García (Hospital del Poniente, El Ejido); Ana Carolina Caballero (Hospital de Zamora); José María Montón (Hospital Obispo Polanco, Teruel); Teresa Mut (Hospital Provincial de Castellón); Eva Manteiga (Hospital Infanta Cristina, Parla); Alejandro de la Serna (Hospital de Galdakao); Ana Esther Trujillo (Hospital General de La Palma); Rafael Blancas (Hospital del Tajo, Aranjuez); Inmaculada Vallverdú (Hospital Universitario San Juan, Reus); José Manuel Serrano (Hospital Universitario Reina Sofía, Córdoba); Miquel Ferrer (Hospital Clinic-IDIBAPS, Barcelona); Juan Diego Jiménez (Hospital de Don Benito); Carlos Gallego (Hospital Infanta Elena, Valdemoro); Dolores Vila (Hospital Meixoeiro, Vigo); Luis Marina (Complejo Hospitalario de Toledo); Valentín Parra (Hospital de Sagunto); Juan Ramón Cortés (Complejo Hospitalario de Ourense).

*Taiwan*:

Chen Chin-Ming & Ai-Chin Cheng (Chi Mei Medical Center, Tainan City)

*Tunisia*:

Coordinator: Fekri Abroug (Hospital Fattouma Bourguina, Monastir)

Besbes Mohamed (Hospital Abderrahmane Mami, Ariana); Imed Chouchene (Hospital Universitarie Farhat Hached, Sousse); Mounir Bouaziz (CHU Habib Bourguiba, Sfax); Stambouli Neji & Islem Ouanes (Hospital Fattouma Bourguina, Monastir); Ayed Samia (Hospital Taher Sfar City :Mahdia).

*Turkey*:

Coordinator: Nahit Cakar (Istanbul Medical Faculty, Istanbul)

Ismail Kati (Medical Faculty of Yuzuncu Yil University, Van) Ali Aydım Altunkan (Faculty of Medicine Mersin University); Remzi Iscimen (Uludag University Faculty of Medicine, Bursa); Zafer Dogan (Sutcu İmam University, Kahramanmaras); Bilge Çetin(Erciye Üniversity); Tayfun Adanir (Atatürk Hospital , Izmir); Sabriye Guvenc (Anadolu Medical Center Istanbul); Unase Büyükkoçak (Kirikkale University).

*United States*:

Coordinator: Antonio Anzueto (University Hospital San Antonio and South Texas Veterans Health Care System)

Ashley Ellis & Gary Kinasewitz (Oklahoma University Health Science Center &VA medical Center, Oklahoma City); Allan Walkey & Phil Alkana; (Boston Medical Center, Boston); Gregory A. Schmidt, Susan Gillen, Kathleen Lilli, Jennifer Twombley, Denice Wells & Larry Welder; (University of Iowa Hospitals and Clinics, Iowa Vity); Alejandro Arroliga, Alfredo Vasquez-Sandoval, Vincent John Scott, Craig Cernosek & Christopher Spradley; (Temple Clinic, Scott & White Healthcare, Temple); Dimple Tejwani, Sindhaghatta Venkatram & Gilda Diaz-Fuentes (Bronx Lebanon Hospital Center, New York); Amber Monson, Anthony Saleh, Madhav Gudi & George Liziamma; (New York Methodist Hospital, New York); Mohamed A Saad, Crissie De Spirito, Bryan Beatty, Samir Vermani & Crissie Despirito; (University of Louisville School of Medicine Hospital, Louisville); Zaza Cohen, Amee Patrawalla, Samir Abdelhadi, Rupesh Vakil & Steven Y. Chang (UMDNJ-New Jersey Medical School, Newark); Brian Sherman, Rosanna Del Giudice & John Oropell (The Mount Sinai Medical Center, New York); Timothy D Girard, Cayce Strength, Joyce Okahashi, Leanne Boehm & Matthew Kirchner (Vanderbilt University School Of Medicine, Nashville); Ashley Ellis & Gary Kinasewitz (Oklahoma City VA Medical Center, Oklahoma City); Erwin J. Oei, Sebastian Circo, Nelson Medina & Mohammed Al-Jagbeer (Morristown Medical Center, Morristown); V.J. Cardenas, Jr & Smyth Smith (University Of Texas Medical Branch at Galveston); Shelby Sutton, Marcela Canola-Mazo, Tim Houlihan, Yogeet Kaur & Travis Parry  (University Hospital San Antonio and South Texas Veterans Health Care System); Craig A. Piquette & Kerry Canady (Omaha VA Medical Center. Omaha); Rahul Nanchal & Dana K. Soetaert (Medical College Of Wisconsin); Maria del Mar Torres-Perez, Carlos Robles-Arias & William Rodriguez-Cintron (VA Caribbean Health Care System, San Juan, Puerto Rico); Mark Tidswell, Jennifer Germain, Lori-Ann Kozikowski & Erin Braden (Baystate Medical Center, Springfield); Geneva Tatem (Henry Ford Hospital Detroit)

*Uruguay*:

Coordinator: Javier Hurtado (Hospital Español & CUDAM, Montevideo)

Alberto Deicas (CASMU Nº2, Montevideo); Daniel Weiss (Hospital Pasteur, Montevideo); Marta Beron (Hospital Maciel, Montevideo); Román Garrido (Hospital Evangélico, Montevideo); Cristina Santos & Mario Cancela (Hospital de Clínicas, Montevideo); Raúl Lombardi (Impasa, Montevideo); Pedro Alzugaray (CAAMOC, Carmelo, Sanatorio Americano, Montevideo & Orameco, Colonia); Jorge Gerez (Hospital Policial, Montevideo); Silvia Mareque (Sanatorio CAMS, Mercedes); Graciela Franca (Circulo Católico, Montevideo); Oscar Cluzet (Sanatorio Americano, Montevideo); Edgardo Nuñez (Sanatorio Mautone & Hospital de Maldonado, Maldonado); Julio Pontet (Hospital de Florida, Florida); Sergio Cáceres (Centro Cardiológico Sanatorio Americano, Montevideo); Elias Caragna (CRAMI, Las Piedras); Alberto Soler (COMEPA & Hospital Escuela del Litoral, Paysandú); Frank Torres (Sanatorio Cantegril, Punta del Este); Gastón Pittini (CAAMEPA, Pando).

*Venezuela*:

Coordinator: Gabriel d`Empaire (Hospital de Clínicas de Caracas)

Stevens Salva & Fernando Pérez (Hospital de Clínicas de Caracas); Clara Pacheco & Zoraida Parra (Hospital Clínico Universitario de Caracas); Ingrid Von der Osten (Hospital Miguel Pérez Carreño, Caracas); Luis Williams & José Salinas (Hospital Centro de Especialidades de Anzoategui).

*Vietnam*:

Do Danh Quynh, Pham Thi Van Anh, Nguyen Huu Hoang & Nghuyen ba Tuan (Viet Duc Hospital, Hanoi).

Investigators in the Fourth International Study on Mechanical Ventilation (2016)

*Argentina*:

Coordinator: Fernando Rios (Hospital Nacional Profesor Alejandro Posadas, Haedo & Sanatorio Las Lomas, San Isidro, Buenos Aires)

Antonio Conforti (Hospital Fiorito, Avellaneda); Ariel Chena, Carlos Pellegrini (Hospital Lagomaggiore, Mendoza); Gustavo Plotnikow (Sanatorio Anchorena, Buenos Aires); Janina Lebus (Clinica La Sagrada Familia Neurological, CABA, Buenos Aires), Javier Osatnik (Hospital Alemán, Buenos Aires); Judith Sagardia (Hospital Nacional Profesor Alejandro Posadas, Haedo); Lisandro Bettini (Hospital Provincial del Centenario, Rosario); Marcela María Ducrey, Nicolás Iezzi (Hospital Italiano, Buenos Aires); Marco Bezzi (Hospital General de Agudos Donación Francisco Santojanni, Buenos Aires); Mónica Edith Emmerich (Sanatorio Güemes, Luján); Nicolás Zuljevic (Hospital San Martin, Buenos Aires); Pablo Edgardo Gómez (Sanatorio Juncal, Buenos Aires); Raúl Alejandro Gómez (Sanatorio Los Arcos, Buenos Aires); Rubén O. Fernández (Hospital Español, Mendoza); Santiago Ilutovich, Sebastián Fredes (Sanatorio Trinidad Mitre, Buenos Aires); Sebastián Mare (Hospital Zonal, Esquel)

*Australia*:

Coordinators: Andrew Bersten; Elisha Matheson (Flinders Medical Centre, Bedford Park)

Helen Rodgers, Frank van Haren, Josie Russell-Brown, Mary Nourse, Katie Jefferson, Sarah Mckew (Canberra Hospital); Helen Wong (Concord Repatriation General Hospital, New South Wales); Kate Schwartz (Flinders Medical Centre, Bedford Park); Sandra Coutts (Ballarat Hospital)

*Belgium*:

Coordinator: Greet Hermans (UZ Leuven)

Marc Bourgeois, Joke Denolf (AZ Sint Jan Brugge, Oostende); Helga Ceunen (UZ Leuven).

*Brazil*:

Coordinator: Bruno Valle-Pinheiro (Hospital Universitário da Universidade Federal de Juiz de Fora, Juiz de Fora).

Gustavo Silva, Cristina Prata (Hospital Pio XII, Barretos); Ana Lúcia Gut (Hospital das Clínicas da Faculdade de Medicina de Botucatu, Botucatu); Arthur Oswaldo Abreu (Clínica São Vicente, Rio de Janeiro), Bruno Bonaccorsi (Hospital Octaviano Neves, Belo Horizonte); Cassiano Teixeira (Hospital Moinhos de Vento, Porto Alegre); Cintia Magalhães (Hospital Evangélico de Londrina, Londrina); Cristiane Andrade (Hospital Júlia Kubitschek, Belo Horizonte); David José Ibrahim Zeghzeghi (Santa Casa de Misericórdia de Belo Horizonte, Belo Horizonte); Fabio Ferreira (Hospital Santa Luzia, Brasília); Edimar Pedrosa (Hospital Doutor João Penido, Juiz de Fora); Fernando Fonseca (Hospital São João Batista, Viçosa); Flávio Luiz de Aguiar (Hospital João XXIII, Belo Horizonte); Frederico Costa (Hospital da Polícia Militar de Belo Horizonte, Belo Horizonte); Hélady Sanders-Pinheiro (Hospital Albert Sabin, Juiz de Fora); Carina Oliveira Freire (Hospital Unimed, Belo Horizonte); Hugo Urbano (Hospital Vila da Serra, Belo Horizonte); Jorge Luiz Rocha (Santa Casa de Misericórdia de São João del Rei, São João del Rei); José Carlos Fernandez Versiani (Hospital Madre Teresa, Belo Horizonte); Juliana Carvalho (Hospital das Clínicas da Universidade de São Paulo); Maria Carmen Mendes (Hospital de Pronto Socorro Risoleta Tolentino Neves, Belo Horizonte); Maria Aparecida Braga (Hospital Dia e Maternidade Unimed, Belo Horizonte); Maycon Moura (Hospital Monte Sinai, Juiz de Fora); Milton Rodrigues Júnior (Hospital São Paulo, São Paulo); Neymar Elias de Oliveira (Hospital de Base de São José do Rio Preto, São José do Rio Preto); Nivaldo Santos (Hospital de Cataguases, Cataguases); Amaury César Jorge (Hospital do Câncer, Uopeccan); Péricles Almeida Delfino (Hospital Universitário do Oeste do Paraná, Cascavel); Rovilson Lara (Hospital Arnaldo Gavazza Filho, Ponte Nova); Rubens Altair Amaral de Pádua (Hospital Vaz Monteiro, Lavras); Sergio Teixeira Sant’Anna (Hospital Norte D’Or, Rio de Janeiro); Thatiane Olivier Ticom (Hospital Márcio Cunha, Ipatinga); Wilson de Oliveira (Hospital Pronto Socorro 28 de Agosto, Manaus).

*Canada*:

Coordinator: Lorenzo del Sorbo (University Health Network, Interdepartmental Division of Critical Care Medicine, University of Toronto).

Tommaso Pettenuzzo, Eddy Fan, Niall D. Ferguson (University Health Network, Interdepartmental Division of Critical Care Medicine, University of Toronto).

*Chile*:

Luis Soto-Germani. (Hospital San Pablo, Coquimbo)

*China*:

Coordinator: Bin Du (Peking Union Medical College Hospital, Beijing).

Yu Chen, Chenming Dong (Lanzhou University 2nd Hospital); Chengzhi Ding, Cheng Nie, Qiang Shao, Yan Zhang (Nanchang University 1st Hospital); Qian Chen (Southwest Medical University Affiliated Hospital); Lei Xu (Tianjin 3rd Central Hospital); Wei Jiang (Peking Union Medical College Hospital, Beijing); Yu Ma (Chongqing Emergency Service Center); Feng Shen (Guizhou Medical University Affiliated Hospital); Feng Sui, Jiahui Miao, Wenxiong Li, Wenliang Ma, Yijia Jiang, Yue Zheng, Qiuchen Zhang (Beijing Chaoyang Hospital); Ying Gao (Tianjin Medical University General Hospital); Jingli Gao (Kailuan General Hospital); Xiaohui Gong (Chifeng Medical College Hospital); Liqing Guo (Shihezi University 1st Hospital); Haibo Qiu (Zhongda Hospital, Southeast University); Jinhai Han (Qinghai Provincial People's Hospital); Hongcheng Tian (China Rehabilitation Center); Hongsheng Zhao (Nantong University Affiliated Hospital); Ming Hou (Qinghai University Affiliated Hospital); Huaicong Long (Sichuan Provincial People's Hospital); Chulin Huang (Cancer Hospital, Chinese Academy of Medical Sciences); Huiqing Ge (Sir Run Run Shaw Hospital, Zhejiang University Medical School); Jian Zhang (Baoding 1st Central Hospital, East Campus); Jian Zhou, Xuefei Yang, Yaoli Wang (Daping Hospital, Research Institute of Surgery, Third Military Medical University); Rui Li (Henan Provincial People's Hospital); Ning Li (Hebei University Hospital); Li Yu, Xiaoling Wu (Wuhan Central Hospital); Liwei Pan (Wenzhou Medical University 2nd Hospital); Deyong Li (Jurong People's Hospital); Lijuan Zhang, Meng Wu (Chengdu 5th People's Hospital); Lina Guan (Yantai Yuhuangding Hospital); Liping He (Inner Mongolia Medical University Hospital); Liquan Huang (Zhejiang Provincial TCM Hospital); ; Lei Liu (PLA Shenyang General Hospital); Mili Liu (Xinjiang People's Hospital); Shuangqing Liu (PLA General Hospital 1st Affiliated Hospital); Jingfeng Liu, Zhide Yuan (Beijing Friendship Hospital, Capital Medical University); Xiujuan Liu, Zhigang Zuo (Qinhuangdao 1st Hospital); Pan Han (Zhejiang University Medical School 2nd Hospital); Jing Pang (Guangxi Provincial People's Hospital); Peijun Li (Tianjin Chest Hospital); Xuehua Pu (Taizhou People's Hospital); Qinggang Ge (Peking University 3rd Hospital); Qiuhui Wang (Wuxi People's Hospital); Ruilan Wang, Yun Xie; (Shanghai 1st People's Hospital); Ruiqiang Zheng (Yangzhou Subei People's Hospital); Wenjuan Shen; Shenzhen Baoan (Nanhua Unviersity 2nd Hospital); Shengyuan Su (Shenzhen Baoan District People's Hospital); Weizheng Shuai (PLA Navy General Hospital); Shuangping Zhao (Xiangya Hospital, Zhongnan University); Yingying Su, Linlin Fan (Xuanwu Hospital, Capital Medical University); Jiajun Sun (Liaocheng 2nd People's Hospital); Yun Sun (Anhui Medical University 2nd Hospital); Tongwen Sun (Zhengzhou University 1st Hospital); Xiaozhi Wang (Binzhou Medical College Affiliated Hospital); Xue Wang (Xi'an Jiaotong University 1st Hospital); Xianyao Wan (Dalian Medical University 1st Hospital); Xiaobo Huang, Xiaohong Zhang (Sichuan Provincial People's Hospital); Xiaomao Xu (Beijing Hospital); Xiaomei Chen (Shandong University Qilu Hospital); Haifeng Xu (Changzhou Wujin Hospital); Yongfang Zhou (West China Hospital, Sichuan University); Yuan Yuan (Gansu Provincial People's Hospital); Yuetian Yu (Renji Hospital, West Campus, Shanghai Jiaotong University Medical School); Yujie Li (Renji Hospital, East Campus, Shanghai Jiaotong University Medical School); Dehou Zhang (Jiangsu University Jiangbin Hospital); Kun Zhang (Hebei Medical University 4th Hospital); Minwei Zhang (Xiamen University 1st Hospital); Qiguang Du (Zhenjiang 1st People's Hospital); Zhenglong Ye (Nanjing Jiangbei People's Hospital); Zhenyi Rao (Kunming Medical University 5th Hospital); Zhiyong Peng (Zhongnan Hospital, Wuhan University); Fachun Zhou (Chongqing Medical University 1st Hospital); Jianxin Zhou (Tiantan Hospital); Yibing Zhu (Shandong Qianfoshan Hospital); Zhang Jun (Suzhou Municipal Hospital, East Campus); Huang Min Qiang (University of Hong Kong Hospital); An Lili (Yingkou Development Zone Central Hospital).

*Colombia*:

Coordinator: Marco González (Clínica Medellín & Universidad Pontificia Bolivariana, Medellín, Medellín).

Adriana Paola Franco, Gineth Viviana Lozano, Daniel Rojas, Carolina Chingate, Leidys Vidales, Mauricio Muñoz, Diana Romero, Bibiana Telag, Taylor Burbano (Clínica Meta, Villavicencio); Angélica María Luna Diana, Angélica Carrillo, Harvey Moncayo, Juan Pablo Ospina (Clínica Medilaser, Neiva), Bladimir Alejandro Gil (Clínica Las Américas, Medellín); Carlos Hurtado (Centro Policlínico & Clinica Los Nogales, Bogotá); Cesar Enciso (Hospital Infantil San José, Bogotá); Carlos Mauro Arias (Clínica Antioquia, Medellín); David Yepes (Clínica CES, Medellín); Federico Andrés Benítez (Clínica La Estancia, Popayán); Francisco Molina (Clínica CUB, Medellín); Mario Gómez, Giovanni Libreros (Clínica Colsubsidio, Bogotá); Gloria Cepeda (Clínica SOMA, Medellín); Guillermo Ortiz (Clínica de Santa Clara, Bogotá); Mario Gómez Duque (Hospital San José, Bogotá); Felipe Álvarez (Hospital San Rafael de Itagui, Medellín); José Fidel Jaraba (Clínica General Del Norte, Barranquilla); Juan Luis Echeverri (Clínica Medellín, Medellín); Lina María Saucedo, (Clínica Shaio, Bogotá); María Elena Ochoa, Marcela Granados (Clínica Fundación Valle de Lili, Cali); Norton Pérez Gutiérrez (Hospital Departamental, Villavicencio); Rafael Thomen (Clínica la Asunción, Barranquilla); Rigoberto Espinosa (Clínica del Prado, Medellín); Ronald Antonio Medina (Umas Móvil Ips, Bogotá); Victoria Eugenia Ángel Mejía, (Hospital Manuel Uribe Ángel, Medellín); Yenny Rocío Cárdenas, Leopoldo Ferrer (Hospital Universitario Fundación Santafé, Bogotá); Rigoberto Espinosa (Clínica del Prado, Medellín)

*Ecuador*:

Coordinator: Manuel Jibaja (Hospital de Especialidades Eugenio Espejo & Escuela de Medicina de la Universidad Internacional del Ecuador, Quito).

Fernanda García, Vanesa Ramírez (Hospital de Especialidades Eugenio Espejo, Quito); Jorge Hurtado (Hospital Carlos Andrade Marín, Quito); Gustavo del Pozo (Hospital General Fuerzas Armadas, Quito); Estuardo Salgado (Clínica la Merced, Quito); Victor Manuel Figueroa (Hospital de SOLCA, Quito) Marcelo Ochoa, Priscilla Reinoso (Hospital José Carrasco Arteaga, Cuenca); Francisco Rivadeneira, Mario Acosta (Hospital San Vicente de Paúl Ibarra).

*El Salvador*:

Manuel Bello (Hospital Nacional San Rafael, Hospital Nacional Zacamil, Hospital Militar, San Salvador).

*France*:

Coordinator: Arnaud W. Thille (Centre Hospitalier Universitaire de Poitiers)

Nadia Anguel, Alexandra Beurton (Centre Hospitalier Universitaire du Kremlin-Bicêtre); Gaëtan Béduneau, Christophe Girault (Centre Hospitalier Universitaire Charles Nicolle, Rouen), Pascal Beuret, Simon Devillez (Centre Hospitalier de Roanne); Florence Boissier (Centre Hospitalier Universitaire de Poitiers); Anne Bretagnol, Armelle Mathonnet (Centre Hospitalier d’Orléans); Audrey de Jong, Samir Jaber (Centre Hospitalier Universitaire Saint Eloi, Montpellier); Jean Dellamonica (Centre Hospitalier Universitaire d’Archet, Nice); Elisabeth Gratia, Nicolas Terzi (Centre Hospitalier Universitaire Grenoble Alpes); Claude Guérin, Sophie Perinel (Hôpital de La Croix Rousse, Lyon); Emmanuel Guérot, Alexandra Monnier (Hôpital Européen Georges Pompidou); Matthieu Le Meur, Damien Roux (Hôpital Louis Mourier, Colombes); Erwan L’Her (Centre Hospitalier Universitaire de Brest); Saad Nseir, Anahita Rouzé (Centre Hospitalier Universitaire de Lille); Thierry Soupison (Centre Hospitalier Universitaire d’Amiens).

*Germany*:

Coordinator: Konstantinos Raymondos (Medizinische Hochschule Hannover).

Johannes Bickenbach, Jessica Pezechk (Universitaetsklinikum, Aachen); Cezar Mihailescu, Thomas Moellhoff  (Katholische Stiftung Marienhospital, Aachen); Roland Schneider, Ronald Matties (Klinikum Bad Salzungen gGmbH, Bad Salzungen); Hansjörg Haas (Ermstalklinik Bad Urach - Kreiskliniken Reutlingen GmbH, Bad Urach); Stefan Seyboth, Volker Schoeffel (Klinikum Mittelbaden Baden-Baden Balg, Baden-Baden); Jorg Reutershan (Klinikum Bayreuth GmbH, Bayreuth); Stefan Weber-Carstens (Charité Universitaetsklinikum, Berlin); Jorg Brederlau (HELIOS Klinikum Berlin Buch, Berlin); Michael Kraemer (Deutsches Herzzentrum, Berlin) Christian Putensen (Universitaetsklinikum, Bonn); Jorg Ahrens (Zentralkrankenhaus Links der Weser, Bremen); Kirsten Terhorst, Alexander Buchmann (KRH Klinikum Großburgwedel, Burgwedel); Ralph Sander (Allgemeines Krankenhaus, Celle); Bodo Albrecht (Zeisigwaldkliniken Bethanien, Chemnitz); Matthias Rabba, Christian Weilbach (St. Josefs-Stift, Cloppenburg); Jens Buettner (Evangelisches Krankenhaus Elisabethenstift gGmbH, Darmstadt); Linda Reisbach, Alin Ulbricht (Universitaetsklinikum Carl Gustav Carus, Dresden); Christian Jung, Ernan Zhu (Universitaetsklinikum, Dusseldorf); Maren Schmidt, Dirk Weiland (Klinikum Barnim GmbH, Werner Forßmann Krankenhaus, Eberswalde); Frank Herbstreit, Jurgen Peters (Universitaetsklinikum, Essen) Jürgen Ernst, Christoph Wiesenack (Evangelisches Diakoniekrankenhaus Freiburg im Breisgau, Freiburg); June Tomelden (ASKLEPIOS Fachkliniken Muenchen-Gauting, Gauting); Stefan Rauch, Matthias Fischer (Klinik am Eichert, Goeppingen); Andrea Kenchen (Universitaetsklinikum der Georg-August-Universitaet, Goettingen); Maja Iversen (Bundeswehrkrankenhaus, Hamburg) Yones Salameh, Wolfgang Seitz (Kreiskrankenhaus, Hameln); Martin Wagner, Mathias Gnielinski (DRK-Krankenhaus Clementinenhaus, Hannover); André Gottschalk, Frauke Honig (DIAKOVERE Friederikenstift, Hannover); Christine Fegbeutel, Wolfgang Knitsch, Mathias Wilhelmi, Heiner Ruschulte, Sebastian Panke (Medizinische Hochschule Hannover, Hannover); Martin Schott, Bernard Tautz (Klinikum Hannover Nordstadt, Hannover); Matthias David, Karin Kobusch (KRH Klinikum Siloah-Oststadt-Heidehaus, Hannover); Hongting Hensel, Jorg Scharnofske (DIAKOVERE Henriettenstift, Hannover); Emanuel Chorianopoulos, Michael Preusch (Universitaetsklinikum der Ruprecht-Karls-Universitaet, Heidelberg); Marion Hoffmann (Thoraxklinik Heidelberg - Universitaetsklinikum der Ruprecht-Karls-Universitaet, Heidelberg); George von Knobelsdorff (St. Bernward Krankenhaus GmbH, Hildesheim); Beate Wedel (Städtisches Klinikum Karlsruhe gGmbH, Karlsruhe); Andreas Hohn, Jochen Hinkelbein, Joanna Plessow, Caroline Rolfes (Universitaetsklinikum, Koeln); Joachim Lindner (KRH Klinikum, Lehrte); Hermann Wrigge (Universitaetsklinikum der Universitaet, Leipzig); Hans F. Ginz (Kreiskrankenhaus, Lörrach); Detlef Goerdes, Christian Frenkel (Städtisches Klinikum, Lueneburg); Caren Tietz (Otto-von-Guericke Universität Universitaetsklinikum, Magdeburg); Walter Pohl (Kliniken Hochfranken, Muenchberg); Christian Siebers, Jochen Henkel, Ludwig Ney, Lorenz Frey (Universitaetsklinikum der Ludwig-Maximilians-Universitaet, München); Chritian Rabe (Klinikum rechts der Isar der TU Muenchen, Abteilung fuer klinische Toxikologie, München); Frank Brettner (Krankenhaus der Barmherzigen Brueder, München); Vanessa Rembold, Joachim Meyer (Staedtisches Krankenhaus Muenchen-Harlaching, München); Michael Ziegler, Karl Schulze (KRH Klinikum, Neustadt am Ruebenberge); Thomas Bein (Universitaetsklinikum, Regensburg); Dirk Holten, Markus Schappacher (Klinikum Sindelfingen-Boeblingen, Sindelfingen); Sebastian Allgaeuer (Robert-Bosch-Krankenhaus, Stuttgart); Tom Phillip Zucker, Martin Glaser (Klinikum Traunstein, Traunstein); Markus Schmola (Kreiskrankenhaus, Woerth an der Donau); Marcus Kredel (Universitaetsklinikum, Wuerzburg); Jean Soukup (Carl-Thiem-Klinikum, Cottbus)

*Greece*:

Coordinator: Dimitrios Matamis (Papageorgiou Hospital, Thessaloniki)

Aikaterini Flevari (Attikon University Hospital, Athens); Christofis Christos (University Hospital, Alexandroupolis); Efstratios Manoulakas (University Hospital, Larissa): Eleni Mouloudi, Eleni Massa (Ippokration Hospital, Thessaloniki); Maria Papaioannou (Papanikolaou Hospital Medical, Thessaloniki); Tasioudis Polychronis (Gennimata Hospital, Thessaloniki); Georgia Vasileiadou, Eleni Synnefaki  (Papageorgiou Hospital, Thessaloniki)

*Guatemala*:

Jorge Luis Ranero, Luis Estuardo Ávila, José Armando Muñoz, Ana Silvia Bonilla, Erick Méndez, Juan José Deyet, Gary Abraham Reyes (Hospital General de Enfermedades, IGSS, Ciudad de Guatemala).

*Ireland*:

Coordinator: Ignacio Martin-Loeches (St James’s University Hospital, Dublin)

David Honan (Wexford General Hospital); Gabor Zilahi (St James’s Hospital, Dublin); Gerry Fitzpatrick (Tallaght Hospital Dublin).

*Italy*:

Coordinator: Salvatore Maurizio Maggiore ().

Agostino Roasio (Presidi Ospedalieri Cardinal Massaia e Vallebelbo, Asti); Alice Sacco (Ospedale Torrette, Ancona); Andrea Bottazzi (Ospedale San Matteo, Pavia); Andrea Neville Cracchiolo (Ospedale Civico, Palermo); Antonella Cotoia (Ospedali Riuniti, Foggia); Camilla Micalizzi (Ospedale Policlinico, Genoa); Federico Longhini (Ospedale Sant'Andrea, Vercelli); Francesca Tardini (Ospedale Niguarda, General ICU, Milano); Francesco Curto (Ospedale Niguarda, Neurosurgical ICU, Milano); Jessica Maugeri (Ospedale ARNAS Garibaldi Centro, Catania); Luca Maria Montini (Policlinico Agostino Gemelli , Università Cattolica Del Sacro Cuore, Roma); Lucia Mirabella (Ospedali Riuniti, Foggia); Marco Pozzi (Ospedale San Matteo, Pavia); Maria Elena (Ospedale San Giovanni Bosco, Torino); Maurizio Bottiroli (Ospedale Niguarda, Cardiosurgical ICU, Milano); Monica Rocco (Policlinico Sant'Andrea, Roma); Nicola Logrieco (Policlinico Federico II, Napoles); Patrizia Murino (Ospedali dei Colli-Monaldi, Napoles); Salvatore Grasso (Ospedale Policlinico, Università Di Bari, Bari); Savino Spadaro (Ospedale Sant'Anna, Ferrara); Sergio Livigni (Ospedale San Giovanni Bosco, Torino); Stefano Romagnoli (Ospedale Careggi, Florence); Tullio Spina (Ospedale Santo Spirito, Pescara).

*India*:

Coordinator: Pravin Amin (Bombay Hospital Institute Of Medical Sciences MS).

Ashwin Kumar Mani (Apollo First Med Hospital, Chennai); Banambar Ray (Apollo Hospitals, Bhubaneswar); Dedeepiya Devaprasad (Apollo Cancer Speciality Hospital, Chennai); Devachandran Jayakumar (Apollo Speciality Hospital OMR, Chennai); Harjit Dumra (Sterling Hospital, Memnagar); Karthiraj Natarajan (Apollo First Med Hospital, Chennai); Leenapatil (Ganpati Hospital); Mehul Shah (Sir. H. N. Reliance Foundation Hospital, Prarthna Samaj); Nagarajan Ramakrishnan (Apollo Hospitals, Critical Care Services, Chennai); R. Ebenezer (Apollo Speciality Hospitals, Chennai); Rajesh Chawla (Indraprastha Apollo Hospitals Sarita Vihar); Samir Sahu (AMRI Hospitals, Bhubaneswar); Suresh Ramasubban (Apollo Gleneagles Hospital, Kolkata).

*Korea*:

Coordinator: Gee Young Suh (Intensive Care and Director of Center for Clinical Epidemiology of Samsung Medical Center, Seoul)

Eumi Jang (Samsung Medical Center, Sungkyunkwan University School of Medicine, Seoul); Heung Bum Lee (Chonbuk National University Medical School and Hospital); Hong Jiyoung (Chuncheon Sacred Heart Hospital, Hallym University Medical Center); Hyung Koo Kang (Ilsan Paik Hospital, Inje University College of Medicine); Jeong Eunsuk (Pusan National University School of Medicine); Ji Young Hong (Chuncheon Sacred Heart Hospital, Hallym University Medical Center); Jin Hwa Lee (Ewha Womans University School of Medicine); Jinhee Jeon (Dongsan Hospital, Keimyung University School of Medicine); Ju Hee Park (Seoul Metropolitan Government Seoul National University Boramae Medical Center); Kim Kyung Chan (Catholic University of Daegu Hospital); Sang-Min Lee (Seoul National University Hospital, Seoul National University College of Medicine); So Young Park (Kyung Hee University Medical Center Seoul); Sunghoon Park (Hallym University Sacred Heart Hospital, Hallym University College of Medicine); Won-Yeon Lee (Yonsei University Wonju Severance Christian Hospital, Yonsei University Wonju College of Medicine); Wonil Choi (Dongsan Hospital, Keimyung University School of Medicine); Yeon Joo Lee (Seoul National University Bundang Hospital); Yun Su Sim (Hallym University Kangnam Sacred Heart Hospital); Chae Man Lim (Asan Medical Center, College of Medicine, University of Ulsan).

*Mexico*:

Coordinator: Maria del Carmen Marin (Hospital Regional 1° de Octubre, Instituto de Seguridad y Servicios Sociales de los Trabajadores del Estado (ISSSTE), México DF)

Abad Q. Ortega Pérez (Centro Médico Lic. Adolfo López Mateos, Toluca); Alfonso Chávez, Ángel Augusto Pérez-Calatayud (Hospital General de México “Dr. Eduardo Liceaga”, México DF); Alfonso García-Luna (Hospital Ángeles, León); Andrea Rugerio (Hospital ABC Santa Fe, México); Alfredo Arellano (Hospital Regional de Alta Especialidad, Ixtapaluca); Arturo Velasco (Hospital La Victoria, Cancún); Asunción José Longino (Hospital Ángeles, Chihuahua); Antonio Tamariz, Christus Muguerza  (Hospital Betania, Puebla); Claudia Ivette Reynoso (Hospital Civil de Guadalajara); Dulce D’ector-Lira (Centro Médico Dalinde, México DF); Damián Gutiérrez-Zarate (Hospital Español, México DF); Dulce Pérez (Hospital Central Universitario, Chihuahua); Eduardo Daniel Anica Malagón (Hospital General de México “Dr. Eduardo Liceaga”, México DF); Enrique Monares (Hospital San Angel Inn Universidad, México DF); Ernesto Deloya (Hospital de Alta Especialidad San Juan del Rio, Querétaro); Ervin Manzo (Hospital Ángeles Metropolitano, México DF); Felipe De Jesús Montelongo (Hospital General Ecatepec “Las Américas”, México DF); Francisco Javier Flores, Mavy Ramírez (Hospital General de Zona No. 1 IMSS, Tepic Nayarit); Gilberto Camarena (Hospital ABC Santa Fe, México DF); Gilberto Felipe Vázquez (Centro Médico Nacional Siglo XXI. Unidad de Trasplante, México DF); Héctor Vázquez (Hospital General de Zona No. 1 del IMSS, La Paz); Gustavo Morales (Hospital Regional de Alta Especialidad de la Mujer, Villahermosa); Sergio Edgar Zamora (Hospital Juárez de México, México DF); Georgina Magaña (Instituto Nacional de Neurología y Neurocirugía MVS. Secretaria de Salud. México DF); Jesús Isidro Sánchez-González (Unidad Médica Alta Especialidad UMAE 34 IMSS, Monterrey); Jorge Rosendo Sánchez-Medina (Hospital Regional de Ciudad Madero Pemex, Tamaulipas); José J Zaragoza (Hospital Ángeles Acoxpa); Juan Antonio Buensuseso (Hospital Costamed Playa del Carmen, Quintana Roo); Juan Carlos Dávila (Hospital General Zona No.1, IMSS. Oaxaca); Julio C Mijangos-Méndez (Hospital Civil de Guadalajara); Manuel Martínez-Medina (Hospital General de Zona No. 5 IMSS, Nogales); María Chacón (INR-CENIAQ. México DF); María Verónica Calyeca (Centro de Especialidades Médicas, Veracruz); José  Jorge Martínez -Soria (Hospital General Irapuato, Irapuato); Raquel Méndez (Hospital Regional 1º de Octubre ISSSTE, México DF); Roberto de Jesús García-Graullera (Hospital Galenia, Cancún); Roberto Rosas (Pemex, Villahermosa); Saira Sanjuana (Hospital Rubén Leñero, México); Sergio Reyes (Hospital General de Cholula, Puebla & UMAE Hospital de Traumatología y Ortopedia, Puebla); Silvio A. Ñamendys-Silva (Instituto Nacional de Cancerología, México DF & Fundación Clínica Médica Sur, México DF); Alfonso Estrada Gutiérrez (Hospital General de la Mujer SSM, Michoacán); Zalatiel Maycotte (Hospital de Texcoco); Villegas Castellanos, Lilian Lizette (Hospital General Ajusco Medio, México DF), Luis Alejandro Sánchez- Hurtado (Hospital de Especialidades “ Dr. Antonio Fraga Mouret” Centro Médico Nacional La Raza. México D.F); Carlos Larios (Hospital de Especialidades del CMN “Manuel Ávila Camacho” del IMSS, Puebla).

*Morocco*:

Coordinator: Amine Ali Zeggwagh (Centre Hospitaler Universitarie Ibn Sina - Mohammed V University, Rabat, Morocco)

Elmostafa Akkaoui (Hôpital Mohamed V, Meknes); Mourad Amor, Hicham Belkhi (Centre Hospitaler Universitarie Ibn Sina, Université Mohamed V, Rabat); Charki Haimeur (Hôpital Militaire d'Instruction Mohamed V, Centre Hospitaler Universitarie Ibn Sina, Rabat); Hanane Ezzouine (Hôpital Ibn Rochd. Casablanca); Mina Elkhayari (Hôpital Ibn Tofail, Centre Hospitalier Universitarie Mohamed V, Marrakech); Tarek Dendane (Hôpital Ibn Sina, Centre Hospitalier Universitarie Ibn Sina, Université Mohamed V, Rabat)

*Netherlands*:

Michael Kuiper & Matty Koopmans (Medical Center Leeuwarden).

*New Zealand*:

Coordinator: Rachael Parke (Auckland City Hospital, Cardiothoracic and Vascular Intensive Care Unit).

Dawn France (Dunedin Hospital); Jennifer Goodson (Tauranga Hospital, Tauranga); Raulle Sol Cruz (Wellington Regional Hospital ICU).

*Nicaragua*:

Becket Arguello (Hospital Central Managua)

*Peru*:

Coordinator: Fernando Rafael Gutierrez-Muñoz (Clínica El Golf, Lima)

Monica Giuliana Meza (Hospital Cayetano Heredia, Lima); José Manuel Cruz (Hospital G. Almenara I,Lima); José Wilber Portugal (Hospital E.Rebagliati M7B, Lima); Manuel Contardo (Clínica San Felipe, Lima); Manuel Laca (Hospital Naval, Lima); Luis Castillo (Instituto de Enfermedades Neoplásicas, Lima); Aland Bisso (Hospital Central de Policía Local, Lima); Rocio Quispe (Clínica Delgado, Lima); Indira Oyanguren (Clínica Angloamericana, Lima).

*Portugal*:

Sofia Dias (Hospital de S. José, Centro Hospitalar de Lisboa) Central, E.P.E; Teresa Honrado (Hospital de S. João, Porto).

*Puerto Rico*:

Gloria Rodríguez, Wilma González-Barreto, Brígida Colón-Barreto, Christian Rosado-Paso, Yara Delgado (HIMA San Pablo Caguas, Puerto Rico).

*Spain*:

Coordinator: Óscar Peñuelas (Hospital Universitario de Getafe, Madrid).

Aaron Blandino, Raúl de Pablo (Medical ICU Hospital Universitario Ramón y Cajal, Madrid); Luis Gajate (Surgical ICU Hospital Universitario Ramón y Cajal, Madrid); Alberto Belenguer (Hospital General de Castellón); Alberto Levy (Hospital Comarcal de Melilla); Alejandro Moneo, Beatriz Busto, Francisco Ortuño (Hospital Clínico San Carlos, Madrid); Alfonso Ortega, Javier Veganzones (Hospital Universitario Puerta de Hierro, Majadahonda); Amparo Cabanillas (Hospital General de Soria); Manuel Cruz, Ana María de Pablo, Susana Zubillaga (Hospital del Sureste, Arganda); Ana Martín-Pellicer (Hospital Rey Juan Carlos, Móstoles); Ana Villagrá (Hospital Txagorritxu, Vitoria); Ángela Algaba, Elena González-González (Hospital de Torrejón); Ángela Alonso-Ovies (Hospital de Fuenlabrada); Antonia Vázquez-Sánchez, Irene Dot, Joan Ramón Masclans (Hospital del Mar, Parc de Salut Mar, Barcelona); Antonio García- Jiménez (Hospital Arquitecto Marcide, Ferrol); Antonio Viñuales (Hospital Lluis Alcanyis, Xativa); Anxela María (Fundación Jiménez Díaz, Madrid); Beatriz Abad, Enrique Cereijo (Hospital Universitario La Princesa, Madrid); Candelaria de Haro (Consorci Hospitalari del Parc Taulí, Sabadell); Caridad Martin- López (Hospital Provincial de Santiago); Carles Subira (Hospital Sant Joan de Déu, Fundació Althaia, Manresa); Carlos Vicent (Hospital Universitario La Fé, Valencia); Carolina García-Martin, Samantha Huidobro (Hospital Universitario de Canarias, La Laguna); Catalina Forteza, Gemma Rialp (Hospital Son Llatzer, Palma de Mallorca); César Laborda, Jordi Riera, Judit Sacanell, Oriol Roca (Hospital Vall d’Hebrón, Barcelona); Concepción Martínez-Fidalgo (Hospital Universitario Infanta Cristina, Parla); David Arizo (Hospital de Sagunt); David Granado (Hospital de Mérida); Pedro J. Domínguez-García (Hospital Juan Ramón Jiménez, Huelva); Cecilia Hermosa (Hospital del Henares, Coslada); Eduardo Morales (Cardiovascular ICU Hospital Clínico San Carlos, Madrid); Eduardo Palencia (Hospital Infanta Leonor, Madrid); Elena Abril, Rebeca Padilla, Fernando Hidalgo (Hospital Universitario de Getafe); Alfonso Estrella (Hospital Universitario de Guadalajara); Eugenio Luis Palazón (Hospital Universitario Reina Sofía, Murcia); Federico Minaya (Hospital San Pedro Alcántara, Cáceres); Félix Martín-González (Hospital Clínico de Salamanca); Fernando Callejo-Torre (Complejo Hospitalario de Burgos); Fernando Iglesias, Manuel Valledor, Marta Martín-Cuadrado (Hospital San Agustín, Avilés); Ferrán Roche-Campo (Hospital de Tortosa); Joan María Raurich, Mireia Ferreruela, María Teresa Millán (Hospital Universitari Son Espases, Palma de Mallorca); Francisco Lucena, Helena Sancho (Hospital de Valme, Sevilla); Germán Escudero (Hospital General Universitario Santa María del Rosell, Cartagena); Gonzalo Hernández (Complejo Hospitalario de Toledo); Francisco Guerrero-López (Complejo Hospitalario Universitario de Granada); Héctor Hernández-Garcés (Hospital de Lliria, Valencia); Hernán Aguirre-Bermeo, María Torrens, Nuria Rodríguez (Hospital de la Santa Creu i Sant Pau, Barcelona); Ignacio Sáez, Susana Temprano, Renata García Gigorro (Medical-Surgical ICU Hospital Universitario Doce de Octubre, Madrid); Isidro Prieto (Trauma ICU Hospital Universitario Doce de Octubre, Madrid); Immaculada Vallverdú (Hospital Universitario Sant Joan de Reus); Iván Astola, Raquel Yano (Hospital Central de Asturias, Oviedo); Jesús Blanco (Hospital Río Hortega, Valladolid); José María Quiroga (Hospital de Cabueñes, Gijón); José Antonio Fernández-Ratero (Neurologic ICU Complejo Hospitalario de Burgos); Miguel Martínez-Barrios (Surgical ICU Complejo Hospitalario de Burgos); José Luis Flordelis, Miguel Ángel Blasco-Navalpotro, Ricardo Díaz-Abad (Hospital Severo Ochoa, Leganés); José María Montón (Hospital Obispo Polanco, Teruel); María Carmen Espinoza (Hospital General Universitario de Ciudad Real); María Elena Plumed, María José Tolon (Hospital Quirón Zaragoza); María Teresa Jurado (Hospital CST Terrasa); María del Carmen Fernández-González (Hospital de Móstoles); María del Rosario Truchero (Hospital Provincial de Ávila); María Elena Pérez-Losada (Hospital Clínico de Salamanca); María Isabel Rubio (Medical-Surgical ICU Hospital Marqués de Valdecilla , Santander); Marta López-Sánchez (Neurologic ICU Hospital Marqués de Valdecilla , Santander); María Isabel Ruiz-García (Complejo Hospitalario de Jaén); María Consuelo Pintado, María Trascasa Muñoz (Hospital Príncipe de Asturias, Alcalá de Henares); Marta Ugalde, Pablo Serna (Hospital de Cruces, Barakaldo); Oscar Martínez-González (Hospital del Tajo, Aranjuez); Miguel León (Hospital Arnau de Vilanova, Lleida); Miquel Ferrer (Respiratory ICU Hospital Clinic, Barcelona); Mònica Magret (Hospital Universitari Joan XXIII, Tarragona); Alfonso Muñoz-López, Roser Tomas, Salvador Fernández (Hospital Carlos Haya, Málaga); Nieves Franco (Hospital de Conxo/Complejo Hospitalario de Santiago de Compostela); Patricia Salgado, Alejandro Suárez-de la Rica (Hospital Universitario La Paz; Madrid); Rafael Sánchez- Iniesta (Complejo Hospitalario Universitario de Albacete); Raquel Montiel (Hospital de la Candelaria, Tenerife); Raquel Montoiro, Carmen Velilla (Hospital Clínico Lozano Blesa, Zaragoza); Raúl Ismael González-Luengo (Complejo Hospitalario de León); Rita Galeiras (Complexo Hospitalario Universitario A Coruña); Rocío Almaraz (Hospital Don Benito-Villanueva de la Serena, Badajoz); Rosa María Álvaro (Hospital de la Plana, Castellón); Roser Tomas (Hospital General de Catalunya, Sant Cugat del Vallès); Sara Cabañes (Hospital Santiago Apóstol, Vitoria); Juan Pedro Tirapu, Manuel García-Montesinos, Adriana García-Herrera, Laura Esther de la Cruz (Complejo Hospitalario Navarra, Pamplona)

*United States of America*:

Coordinator: Antonio Anzueto (University of Texas, Medicine San Antonio, University Hospital San Antonio, San Antonio, Texas); Alexander G Duarte, Shawn Nishi, Shawn  Goodlett, Paula Skinkis, Roxana Hirst (University of Texas Medical Branch, Galveston, Texas); Caryn O. Pope (Baylor College of Medicine - Ben Taub General Hospital, Houston, Texas); Gregory A. Schmidt, Julie A. Amendola (University of Iowa, Iowa City, Iowa); Mayur B. Patel, Morgan A. Hosay, James W. Stewart (Vanderbilt University Medical Center, Nashville, Tennessee); Nikhil Meena (University of Arkansas for Medical Sciences, Little Rock, Arkansas); Abhijit Duggal, Eduardo Mireles-Cabodevila, Gurpreet Kaur, Andrei Hastings, Michelle Ferrari (Cleveland Clinic Foundation and Lerner College of Medicine of Case Western Reserve University, Cleveland, Ohio); Ginger Rivera-Arzon, Felicita Aruz, Yomayra Otero-Dominguez, William Rodriguez-Cintron (San Juan VA Medical Center, San Juan, Puerto Rico); Sheila Habib, Rodolfo Estrada, Hebatallah Assal (University of Texas, Medicine San Antonio, University Hospital San Antonio, San Antonio, Texas)

*Thailand*:

Coordinator: Yuda Sutherasan (Faculty of Medicine Ramathibodi Hospital, Mahidol University, Bangkok).

Napplika Kongpolprom (Faculty of Medicine, King Chulalongkorn Memorial Hospital Chulalongkorn University, Thai Red Cross, Bangkok); Nuttapol Rittayamai Phunsup (Wongsurakiat, Siriraj Hospital Respiratory Care Unit, Department of Medicine, Faculty of Medicine Siriraj Hospital, Mahidol University, Bangkok); Preecha Thomrongpairoj (Siriraj Hospital Medical ICU, Department of Medicine, Faculty of Medicine Siriraj Hospital, Mahidol University, Bangkok); Pongdhep Theerawit (Faculty of Medicine Ramathibodi Hospital, Mahidol University, Bangkok).

*Tunisia*:

Coordinator: Fekri Abroug (Hospital Fattouma Bourguina, Monastir)

Amira Jamoussi (Centre Hopitalier Universitaire Abderrahmane Mami, Ariana); Cherif Mohamed Ali (Centre Hopitalier Universitaire Habib Thameur, Tunis); Ferjani Mustapha (Hôpital Principal d’Instruction Militaire, Tunis); Tayeb Guizani (Centre Hopitalier Universitaire Sahloul, Sousse); Tilouch Nejla (Centre Hopitalier Universitaire Tahar Sfar Mahdia, **Mahdia)**; Zeineb Hammouda Abed (Centre Hopitalier Universitaire Fatouma Bourguiba, Monastir); Zouheir Jerbi (Centre Hopitalier Universitaire Habib Thameur, Tunis).

*Turkey*:

Coordinator: Nahit Cakar (Mehmet Ali Aydınlar Acıbadem Medical Faculty Anesthesiology & Intensive Care, Istanbul)

Başar Erdivanl (Recep Tayyip Erdoğan University Medical Faculty, Anesthesiology & Reanimation, Surgical ICU, Rize); Ahmet Dilek (19 Mayıs University Medical Faculty Mikail Yüksel); Aliye Esmaoglu (Erciyes University Medical Faculty Anesthesiology & Reanimation, Kayseri); Asu Ozgültekin (Haydarpasa Numune Teaching & Research Hospital, Istanbul); Betul Sen (Istanbul Medeniyet University Anesthesiology & Reanimation); Bulent Gucyetmez ( Acibadem International General ICU Istanbul); Cem Erdogan (Medipol University Medical Faculty Anesthesiology & Reanimation Unit Postoperative ICU, Istanbul); Demet Tok (Celal Bayar University Anesthesiology & Reanimation, Reanimation Unit, Manisa); Dilek Surav (Ankara Medicana Hospital, Medical-Surgical ICU, Ankara); Erdogan Ozturk, Hayrettin Daskaya (Bezmialem Vakif University Medical Faculty Anesthesiology & Reanimation General ICU, Istanbul); Evren Senturk (Koç University Medical Faculty, Istanbul); Eyyup Sabri Ozden (Ankara Memorial Hospital, Medical ICU, Ankara); Figen Esen (Istanbul University Istanbul Medical Faculty Emergency Surgical ICU); Lale Karabıyık (Gazi University Medical Faculty Anesthesiology & Reanimation, Ankara); Guldem Turan (Fatih Sultan Mehmet Research & Training Hospital, Istanbul); Hilal Ayoğlu (Bülent Ecevit University Medical Faculty, Zonguldak); Hulya Sungurtekin (Pamukkale University Medical Faculty, Denizli); Hulya Ulusoy (KTU Medical Faculty, Reanimation Unit, Trabzon); Ibrahim Ozkan Akinci (Istanbul University Medical Faculty Neurological ICU); Mehtap Selçuk (Acibadem Kadiköy General ICU Istanbul); Nur Baykara (Kocaeli University Medical Faculty Anesthesiology and Intensive Care, Kocaeli); Perihan Ergin Ozcan (Istanbul University Medical Faculty Medical-Surgical ICU); Ramazan Coskun (Erciyes University Medical Faculty Medical-Surgical ICU, Kayseri); Sedef Tavukçu Özkan (Istanbul Memorial Hizmet Hospital, General ICU); Selcuk Dascı (Acibadem Atakent General ICU, Istanbul); Sema Turan (Ankara Yuksek Ihtisas Research and Training Hospital, General ICU); Serhat Konar (Acibadem Maslak General ICU, Istanbul); Taner Ljama (Acibadem Taksim Hospital General ICU, Istanbul); Umut Acar (Istanbul Florence Nightingale Hospital Surgical ICU); Yavuz Orak (Sivas Numune Public Hospital, Reanimation Unit, Sivas); Yuksel Ela (Afyon Kocatepe University Anesthesiology & Reanimation).

*Uruguay*:

Coordinators: Nicolás Nin¸ Alberto Deicas, Carolina Serra (CASMU Montevideo)

Edgardo Nuñez, Patricia Pereira (Hospital Público Elbio Rivero, Maldonado); Lucia Illardi (Sanatorio Mautone, Maldonado); Julio Cabrera, Gastón Burghi, Ignacio Aramendi (Cenaque, Montevideo); Miguel Alfonso, Ariel Bango (Asociación Médica de San José); Arturo Briva, Cristina Santos (Hospital de Clínicas, Montevideo); Elizabeth Montoya (Casa de Galicia, Montevideo); Alvaro Giordano (Hospital de Florida); Richard Pais, Nicolas Nin, Javier Hurtado (Hospital Español), Lucía López, Diego Mendez, Carlos Nuñez (AESM, Montevideo); Gustavo Pittini (CAAMEPA, Pando, Sauce).
